# Activin A levels are raised during human tuberculosis and blockade of the activin signaling axis influences murine responses to *M. tuberculosis* infection

**DOI:** 10.1128/mbio.03408-23

**Published:** 2024-02-20

**Authors:** Natalie E. Nieuwenhuizen, Geraldine Nouailles, Jayne S. Sutherland, Joanna Zyla, Arja H. Pasternack, Jan Heyckendorf, Björn C. Frye, Kerstin Höhne, Ulrike Zedler, Silke Bandermann, Ulrike Abu Abed, Volker Brinkmann, Birgitt Gutbier, Martin Witzenrath, Norbert Suttorp, Gernot Zissel, Christoph Lange, Olli Ritvos, Stefan H. E. Kaufmann, the CAPNETZ Study group, the DZIF TB study group

**Affiliations:** 1 Department of Immunology, Max Planck Institute for Infection Biology, Chariteplatz, Berlin, Germany; 2 Institute for Hygiene and Microbiology, Julius Maximilian University of Würzburg, Würzburg, Germany; 3 Department of Infectious Diseases, Respiratory Medicine and Critical Care, Charité – Universitätsmedizin Berlin, corporate member of Freie Universität Berlin, Humboldt-Universität zu Berlin, and Berlin Institute of Health, Berlin, Germany; 4 Vaccines and Immunity Theme, Medical Research Council Unit The Gambia at the London School of Hygiene and Tropical Medicine, Fajara, The Gambia; 5 Department of Data Science and Engineering, Silesian University of Technology, Gliwice, Poland; 6 Department of Physiology, Faculty of Medicine, University of Helsinki, Helsinki, Finland; 7 Department of Medicine I, University Hospital Schleswig-Holstein, Kiel, Germany; 8 Department of Pneumology, Clinic, Medical Center, University of Freiburg, Faculty of Medicine, University of Freiburg, Freiburg, Germany; 9 Microscopy Core Facility, Max Planck Institute for Infection Biology, Chariteplatz, Berlin, Germany; 10 CAPNETZ STIFTUNG, Hannover, Germany; 11 German Center for Lung Research (DZL), Berlin, Germany; 12 Division of Clinical Infectious Diseases, Research Center Borstel, Borstel, Germany; 13 German Center for Infection Research (DZIF), Partner Site Hamburg-Lübeck-Borstel-Riems, Borstel, Germany; 14 Respiratory Medicine and International Health, University of Lübeck, Lübeck, Germany; 15 Baylor College of Medicine and Texas Children´s Hospital, Global TB Program, Houston, Texas, USA; 16 Max Planck Institute for Multidisciplinary Sciences, Emeritus Group Systems Immunology, Göttingen, Germany; 17 Hagler Institute for Advanced Study, Texas A&M University, College Station, Texas, USA; Washington University in St. Louis, St Louis, Missouri, USA

**Keywords:** activin A, ActRIIB, tuberculosis, latent TB infection, pneumonia, sarcoidosis, CD103, treatment monitoring, T_RM_, resident memory T cells, IP-10

## Abstract

Activin A strongly influences immune responses; yet, few studies have examined its role in infectious diseases. We measured serum activin A levels in two independent tuberculosis (TB) patient cohorts and in patients with pneumonia and sarcoidosis. Serum activin A levels were increased in TB patients compared to healthy controls, including those with positive tuberculin skin tests, and paralleled severity of disease, assessed by X-ray scores. In pneumonia patients, serum activin A levels were also raised, but in sarcoidosis patients, levels were lower. To determine whether blockade of the activin A signaling axis could play a functional role in TB, we harnessed a soluble activin type IIB receptor fused to human IgG1 Fc, ActRIIB-Fc, as a ligand trap in a murine TB model. The administration of ActRIIB-Fc to *Mycobacterium tuberculosis*-infected mice resulted in decreased bacterial loads and increased numbers of CD4 effector T cells and tissue-resident memory T cells in the lung. Increased frequencies of tissue-resident memory T cells corresponded with downregulated T-bet expression in lung CD4 and CD8 T cells. Altogether, the results suggest a disease-exacerbating role of ActRIIB signaling pathways. Serum activin A may be useful as a biomarker for diagnostic triage of active TB or monitoring of anti-tuberculosis therapy.

**IMPORTANCE:**

Tuberculosis remains the leading cause of death by a bacterial pathogen. The etiologic agent of tuberculosis, *Mycobacterium tuberculosis*, can remain dormant in the infected host for years before causing disease. Significant effort has been made to identify biomarkers that can discriminate between latently infected and actively diseased individuals. We found that serum levels of the cytokine activin A were associated with increased lung pathology and could discriminate between active tuberculosis and tuberculin skin-test-positive healthy controls. Activin A signals through the ActRIIB receptor, which can be blocked by administration of the ligand trap ActRIIB-Fc, a soluble activin type IIB receptor fused to human IgG1 Fc. In a murine model of tuberculosis, we found that ActRIIB-Fc treatment reduced mycobacterial loads. Strikingly, ActRIIB-Fc treatment significantly increased the number of tissue-resident memory T cells. These results suggest a role for ActRIIB signaling pathways in host responses to *Mycobacterium tuberculosis* and activin A as a biomarker of ongoing disease.

## INTRODUCTION

Tuberculosis (TB) is a disease of the lower respiratory tract caused by the intracellular pathogen, *Mycobacterium tuberculosis* (*Mtb*). With 10–11 million new cases and 1.3 million deaths in 2022, it remains one of the most threatening infectious diseases globally (1). To reduce the number of TB cases worldwide, current research focuses on improving diagnosis, anti-TB therapy, and vaccination. Numerous cytokines have been investigated for their role in TB disease and their usefulness in diagnostic biomarker panels but the cytokine activin A has not yet been examined in either context.

Activin A is a member of the TGF-beta (β) superfamily, a group of cytokines that signal *via* heterodimeric receptor complexes composed of two serine-threonine kinases, type I and type II (2, 3). This receptor complex activates receptor-regulated Smads (r-Smads) by phosphorylation, causing them to translocate to the nucleus in association with co-Smad 4 to initiate gene transcription (2, 4). Different functions of TGF-β superfamily members are achieved by the combinatorial diversity of type I and II receptors in the receptor complexes, and the interaction of Smads with multiple transcription factors, co-activators, and co-repressors, as well as through signaling through non-canonical pathways (5). Responses are therefore triggered in a context-specific manner. Activins signal through the type I receptors activin-like kinase (ALK)-4 and -7 and type II receptors ActRIIA and ActRIIB *via* the phosphorylation of Smad2 and Smad3 (2, 6). In addition, non-canonical signaling can occur *via* the phosphatidylinositol 3′-kinase (PI3K)/Akt, MAPK/ERK, and β-catenin/p300 pathways (7–9).

Activin A is highly conserved among mammals, with 100% amino acid identity between humans and mice (2). It is important in the regulation of immune responses, and the first indications that it may play a role in infectious diseases came from studies showing its elevated levels during bacterial sepsis and meningitis (2, 3, 10). Activin A is one of the earliest cytokines released in response to lipopolysaccharides, and neutrophils stimulated with TNF-alpha release pre-formed activin A within 1 hour (2, 11–13). Activin A is produced constitutively by many cell types, but its levels rise in response to disrupted homeostasis (8). Innate immune cells, including monocytes, macrophages, DCs, neutrophils, and NK cells, are thought to be the most important sources of activin A but it can also be produced by T and B cells. The expression of activin A is increased during asthma and acute respiratory distress syndrome (ARDS) (14–16). Recently, it was shown that activin A levels are increased during COVID-19 and are associated with the severity of the disease (17, 18). Like TGF-β, activin A is a multi-faceted molecule with both pro- and anti-inflammatory effects (2, 3). Harmful as well as protective roles for activin A have been observed in respiratory pathology (14, 19–21). It can promote the development of Foxp3^+^ regulatory T cells (T_reg_) and FoxP3^-^ IL-10 producing type I regulatory T cells (T_reg1_) (22, 23) and is implicated in tissue repair processes (24). However, overexpression of activin A in mice leads to respiratory pathology similar to human ARDS and it may play a role in airway remodeling and fibrosis (14, 20, 21, 25, 26).

Due to strong indications that activin A is involved in respiratory pathology, we aimed to determine whether activin A levels were changed during pulmonary TB, as well as during pneumonia and sarcoidosis, diseases that can have a similar clinical presentation and are relevant for differential diagnosis. We also aimed to explore the functional role of activin signaling pathways in TB, using a murine model of TB. Activin A-deficient mice die within 24 hours of birth, and activin receptor type IIA-deficient mice have reproductive deficiencies (27). However, activin A functions have been examined experimentally by overexpression studies, or inhibition with follistatin, receptor inhibitors, or silencing RNAs (3, 28). In our study, we used an inhibitory soluble form of the activin receptor IIB fused to the Fc portion of human IgG1 (ActRIIB-Fc) to block activin signaling pathways in a mouse model of TB. Our results demonstrate that serum activin A levels correlate with the severity of human TB. Furthermore, our preliminary data indicate that ActRIIB signaling pathways influence murine immune responses to *Mtb* infection.

## RESULTS

### Serum activin A levels are raised in active TB

**Fig 1:**
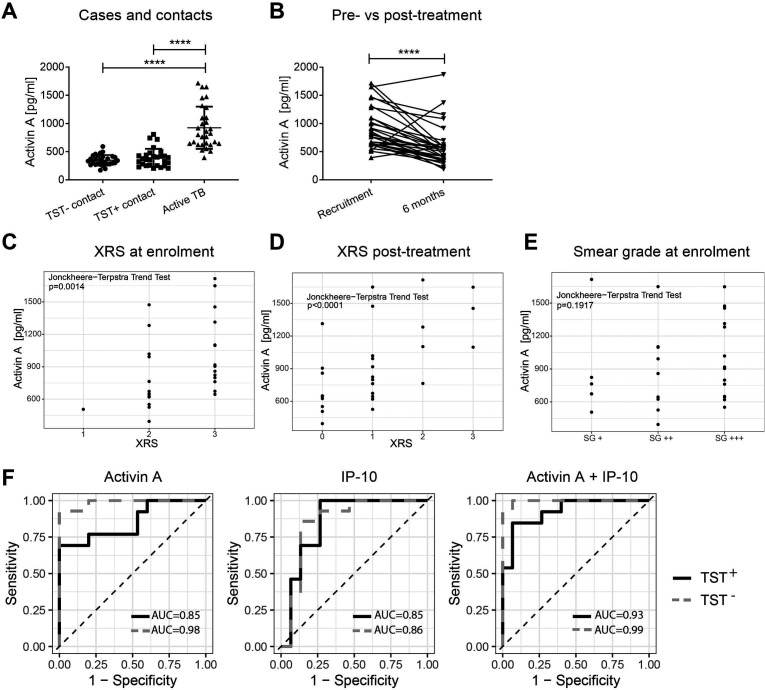
Serum activin A levels in patients with active TB (Gambian cohort). Activin A was measured by ELISA. All patients and controls had black African ethnicity. (A) Serum activin A levels in TB patients at recruitment (*n* = 30) compared to TST- (*n* = 29) and TST+ (*n* = 27) household contacts. Statistical significance was calculated using the Kruskal-Wallis test with Dunn’s multiple comparison test. ****, *P* < 0.0001. (B) Paired serum activin A levels in patients with TB at recruitment and after 6 months of anti-TB therapy. Statistical significance was calculated using the Wilcoxon matched pairs signed rank test. (C) Activin A levels versus X-ray score (XRS) of the cases at recruitment. XRS: 0, normal; 1, minimal disease; 2, moderate disease; 3, advanced disease. Two independent physicians scored the X-rays. (D) Activin A levels versus X-ray score (XRS) of the cases 6 months post-treatment (see C). (E) Activin A levels versus smear grade (SG) of the patient at recruitment. SG: +, 10–99 acid-fast bacilli (AFB) in 100 fields; ++, 1–10 AFB per field; +++, more than 10 AFB per field. For (C, (D, and E), the Jonckheere Terpstra trend test was used to calculate if there was a statistically significant trend between variables. (F) The logistic regression model was used to determine the ability of activin A levels, IP-10 levels or activin A and IP-10 levels in combination to discriminate TB from TST^-^ and TST^+^ healthy contacts. For the obtained models, the receiver operating characteristics (ROC) were calculated together with the area under the curve (AUC).

Serum activin A levels were measured in Gambian TB patients at recruitment and 6 months post-treatment, as well as in healthy tuberculin-skin test negative (TST^-^) and TST^+^ contacts, the latter being a heterogeneous population which may include people who are infected but healthy (who may progress to disease), as well as those who have cleared infection (29, 30). Full details of the Gambian TB cohort are in Table 1. Serum activin A was significantly higher in patients with active TB than in the healthy TST^-^ and TST^+^ controls (*P* < 0.0001) (Fig. 1A). Serum activin A concentrations were diminished in 90% of patients by 6 months post-treatment, with all but three patients showing decreasing serum Activin A after treatment (Fig. 1B). Within 2 years of recruitment, four of the healthy TST^+^ contacts progressed to active TB, diagnosed at days 174, 494, 529, and 671. Only the individual diagnosed with TB at day 174 post-recruitment had raised activin A levels at recruitment (808 pg/mL). Serum activin A levels were higher in patients with a higher X-ray score (XRS) grading of disease severity, both at recruitment (Fig. 1C) and post-treatment (Fig. 1D); therefore, serum activin A levels most likely reflect inflammation in the lungs or severity of disease. The Jonckheere-Terpstra test did not find a significant trend between smear grades and activin A levels (Fig. 1E). A chi-square independence test showed that there was no significant relationship between the smear grades and XRS (*P* = 0.389). In addition to activin A, 27 other serum cytokines and chemokines were measured using multiplex assays, and tested for their ability to discriminate patients with active TB and healthy TST^+^ contacts. IP-10 and IL-9 levels were raised in those with active TB compared to healthy TST^+^ contacts (*P* = 0.004; *P* = 0.011) (Fig. S1A), while IP-10 and IL-1Rα levels were decreased after TB treatment (*P* = 0.030; *P* = 0.029) (Fig. S1B). There were no significant differences in the other cytokines measured (data not shown).

**TABLE 1:** Clinical characteristics and serum activin A levels of the Gambian TB cohort^*a*^

Characteristic	TST-negative contacts	TST-positive contacts	TB cases at recruitment	TB cases at 6 months post-treatment
	(*n* = 29)	(*n* = 27)	(*n* = 30)	(*n* = 30)
Median age, years (IQR)	27 (20–42)	29 (23–39)	27 (21–34)	27 (21–34)
Sex, male/female, n	6/23	13/14	15/15	15/15
Smear grade, n				
–	–	–	0	28
+	–	–	6	0
++	–	–	9	0
+++	–	–	15	0
X-ray score, n				
0	24	18	0	9
1	3	7	1	14
2	2	1	13	4
3	0	0	15	3
Median activin A levels, pg/mL (IQR)	332 (291–410)	376 (265–433)	811 (644–1101)****	532 (381–593)**

^*a*^ The study cohort comprised TB patients as well as TST− and TST+ contacts. Serum samples and clinical data were collected from patients at recruitment and 6 months post-treatment. Of the original 30 individuals per group, one TST− contact and two TST+ contacts were excluded from analysis due to pregnancy. One TST+ contact was excluded because of a diagnosis of TB based on clinical symptoms on Day 1 post-recruitment. X-ray scores and post-treatment smear grades were not available from all individuals. X-ray score: 0, normal; 1, minimal disease; 2, moderate disease; 3, advanced disease. Smear grade: −, negative; +, 10-99 acid-fast bacilli (AFB) in 100 fields; ++, 1-10 AFB per field; +++, more than 10 AFB per field. Serum activin A levels were significantly increased in TB patients at recruitment and 6 months post-treatment compared to TST− and TST+ contacts (**P < 0.01, **** *P* < 0.0001). IQR: interquartile ratio.

### Serum activin A and IP-10 levels can discriminate active TB patients from healthy TST^+^ controls

We performed receiver operating characteristic (ROC) curve analyses to assess the ability of activin A levels, IP-10 levels, and a combination of activin A and IP-10 levels to discriminate patients with active TB disease from the TST^−^ household contacts and those who were TST^+^ (with possible latent TB infection) (Fig. 1F). Serum activin A levels could discriminate those with active TB from both TST^−^ and TST^+^ contacts (see Table S1). There was a positive correlation between levels of serum activin A and IP-10 (*P* < 0.0001, R^2^ = 0.395) (Fig. S1C). IP-10 levels were similarly effective at discriminating between patients with active TB from healthy TST^+^ contacts (see Table S1) but not as effective as activin A levels in discriminating TST^-^ healthy controls from active TB patients. The combination of activin A and IP-10 raised the AUC from 0.85 to 0.93 but was not significantly better in discriminating individuals with active TB from healthy TST^+^ controls than activin A alone (*P* = 0.100) (Fig. 1C; Table S1). Overall, in Gambian patients, activin A shows promise as a biomarker for active TB.

### Serum activin A levels correspond to increased X-ray scores and smear grades

**Fig 2:**
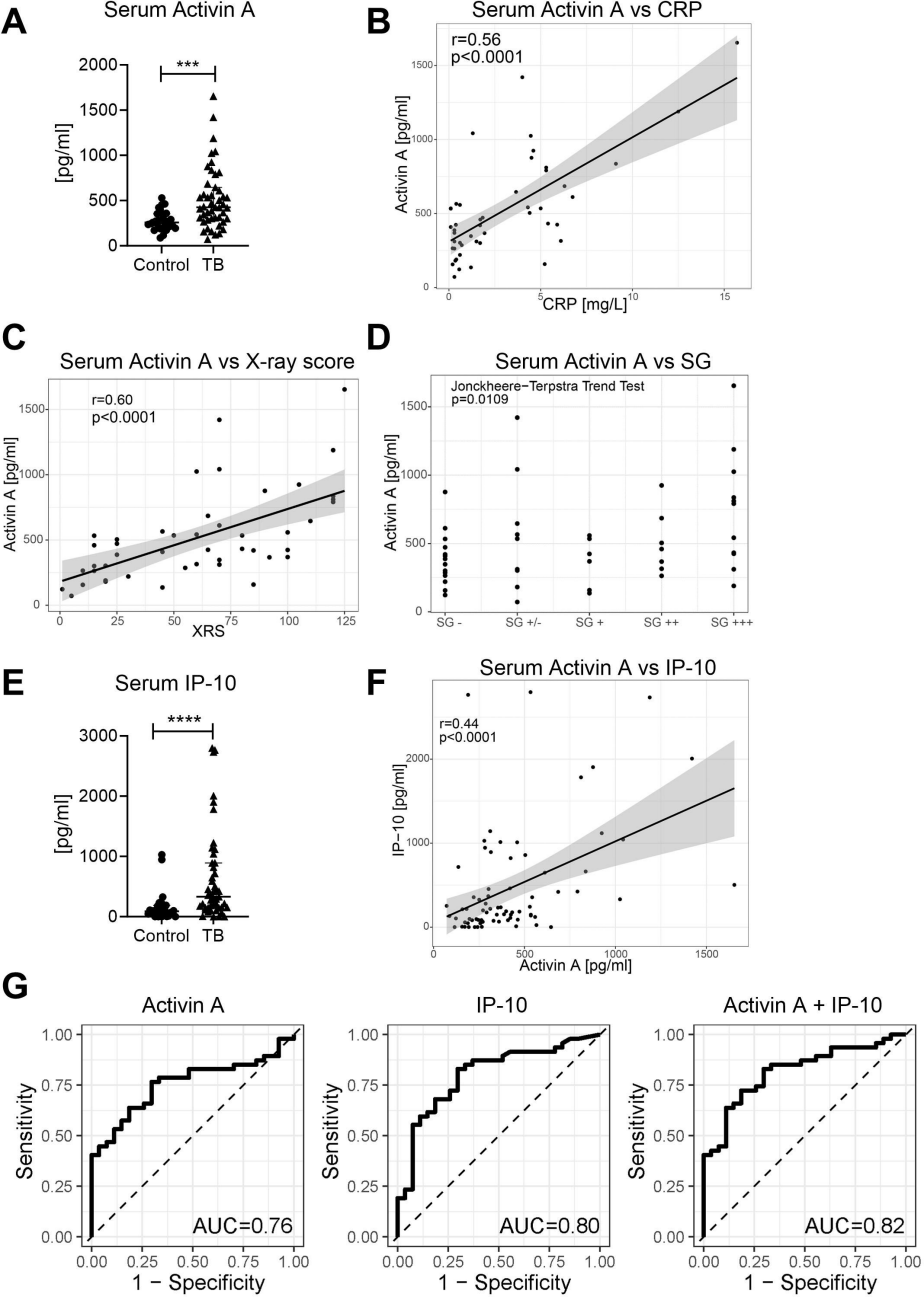
Serum activin A levels in patients with active TB (German cohort). Activin A was measured by ELISA. All controls and the majority of patients had Caucasian ethnicity. (**A**) Serum activin A levels in TB patients at recruitment (*n* = 47) compared to healthy controls (27). Statistical significance was calculated using the Mann-Whitney test. (**B**) Correlation between serum activin A and CRP levels, together with linear regression. (**C**) Correlation between serum activin A levels and XRS (Ralph score, see Methods), together with linear regression. Two independent physicians scored the X-rays. (**D**) Activin A levels versus smear grade (SG) of the patient. The Jonckheere Terpstra trend test was used to calculate if there was a statistically significant trend between SG and activin A levels. SG: -, negative; ±, scanty (1–9 AFB in 100 fields); +, 10–99 AFB in 100 fields; ++, 1–10 AFB per field; +++, more than 10 AFB per field. (**E**) Serum IP-10 levels in TB patients at recruitment (*n* = 47) compared to healthy controls (27). Statistical significance was calculated using the Mann-Whitney test. (**F**) Correlation between serum activin A and IP-10 levels, accompanied by linear regression. (**G**) The logistic regression model was used to determine the ability of activin A levels, IP-10 levels or activin A and IP-10 levels in combination to discriminate TB from healthy contacts. For the obtained models, the receiver operating characteristics (ROC) were calculated together with the area under the curve (AUC).

To validate our findings of increased levels of serum activin A in TB, we examined an independent cohort of TB patients, from Germany. A full description of this cohort is in Table 2. It differed from the Gambian cohort in that not all TB patients were smear positive, though all had TB confirmed by a positive sputum and sputum culture PCR (Xpert). The average age of the German patients and the proportion of males to females was higher (Table S2). Furthermore, while all Gambian patients had drug-sensitive TB, the German patients had an assortment of drug-sensitive, multi-drug resistant (MDR) and extensively drug-resistant (XDR) TB. Despite these differences, serum activin A levels were increased in TB patients compared to controls in the German cohort (Fig. 2A), confirming our finding in the Gambian cohort. However, activin A levels were substantially lower in German patients than in Gambian patients (*P* < 0.0001), with a median of 425 pg/mL compared to 811 pg/mL in Gambian patients (Table S2). While in the Gambian cohort, the control individuals were sub-divided into TST^−^ and TST^+^, in the German cohort the control individuals were a heterogeneous group of IGRA^−^, IGRA^+^, and untested individuals. Activin A levels tended to be higher in the IGRA^+^ controls than in the IGRA^−^ controls but sample numbers were too low to determine whether this was significant (Fig S2A).

**TABLE 2:** Clinical characteristics and serum activin A levels of the German TB cohort^*a*^

Characteristic	Healthy controls	TB cases at recruitment
	(*n* = 27)	(*n* = 47)
Median age, years (IQR)	42 (32–51)	42 (28–47)
Sex, male/female, n	11/16	31/16
Smear grade, n		
–	–	14
±	–	8
+	–	7
++	–	7
+++	–	11
X-ray score, n		
0 (RS 0)	–	0
1 (RS <20)	–	11
2 (RS 21–59)	–	10
3 (RS >60)	–	26
IGRA/ELISPOT, n		
Positive	4	11
Negative	8	1
Not done	15	35
Resistance, n		
Susceptible	–	18
MDR	–	19
XDR	–	10
Median activin A levels, pg/mL (IQR)	257 (205–342)	425 (302–629)***

^*a*^ The study cohort comprised pulmonary TB patients and healthy controls. Serum samples and clinical data were collected from patients at recruitment. Ralph scores (RS) were recorded for the German TB patients (% lung affected plus 40 points if cavities were present). Ralph scores were converted to a 1–3 scale as follows for comparison with the Gambian cohort scores: <20: minimal disease, X-ray score 0; 21–59: moderate disease, X-ray score 1; >60: advanced disease, X-ray score 3. Smear grade: −, negative; ±, scanty (1–9 AFB in 100 fields); +, 10-99 AFB in 100 fields; ++, 1-10 AFB per field; +++, more than 10 AFB per field. Median serum activin A levels were raised in cases compared to controls (***P < 0.001). MDR, multiple drug resistant; XDR, extremely drug resistant.

In the German TB patients, we found a positive correlation between serum activin A levels and the general inflammation marker CRP (Fig. 2B). CRP levels were not measured in the Gambian cohort. There was also a positive correlation between X-ray scores (Fig. 2C) and activin A levels. In this study, the Ralph score (31) was used to assess lung pathology. This score is calculated from the percentage of the affected lung, with an extra 40 points if a cavity is present. In this cohort of TB patients, there was a trend toward increased activin A levels at higher smear grade levels (Fig. 2D) as well as a trend toward higher XRS with higher smear grades (Fig. S2B). Overall, the data suggest that activin A levels are a potential biomarker for the severity of TB disease.

As in the Gambian patients, IP-10 levels were elevated in the German TB patients compared to healthy controls (Fig. 2E) and there was a positive correlation between activin A and IP-10 levels (Fig. 2F). ROC curve analysis demonstrated that serum activin A levels were less able to discriminate between TB patients and healthy controls in the German cohort, with an AUC of 0.76 (*P* < 0.0001) (Fig. 2G). When we stratified the patients according to TB subtype (drug sensitive, MDR or XDR), we found the greatest variance in the drug-sensitive TB group in this cohort (Fig. S2C and D). Activin A levels were significantly higher in the drug-resistant TB groups only (Fig. S2C), while IP-10 was significantly higher in the drug-sensitive TB group (Fig. S2C). For drug-sensitive TB, the combination of activin A and IP-10 could better discriminate TB than activin A alone (*P* = 0.024) (Fig. S2E; Table S1). For MDR and XDR TB, measuring IP-10 did not add value (MDR *P* = 0.808, XDR *P* = 0.889). These findings emphasize the importance of validating biomarker results across different regions and different TB subtypes.

### Serum activin A levels in German pneumonia and sarcoidosis cohorts

**Fig 3:**
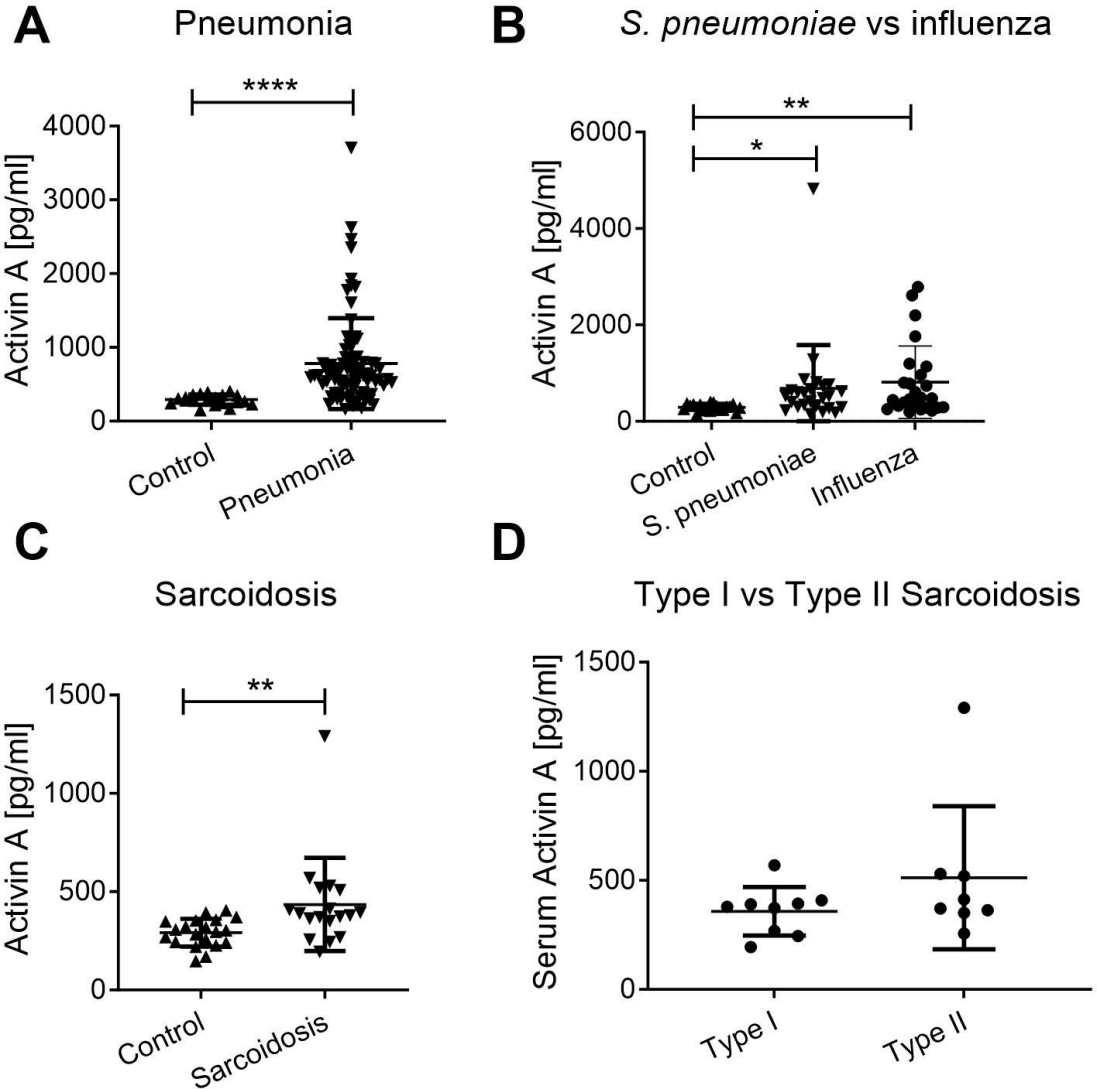
Serum activin A levels in patients with pneumonia or sarcoidosis. Activin A was measured by ELISA. (**A**) Serum activin A levels in pneumonia patients (*n* = 80) versus healthy controls (*n* = 20). (**B**) Serum Activin A levels in selected groups of pneumonia patients in whom *Streptococcus pneumoniae* or Influenza virus were the sole cause of the disease (*n* = 25 per group). (**C**) Serum activin A levels in sarcoidosis patients (*n* = 18) and healthy controls (*n* = 20). Control samples were those collected for the pneumonia study. (**D**) Serum activin A levels in sarcoidosis patients stratified according to X-ray type. Type I (*n* = 9): bilateral hilar adenopathy; Type 2 (*n* = 8): additionally, involvement of pulmonary parenchyma. Statistical significance was calculated using the Mann-Whitney test or the Kruskal-Wallis test with Dunn’s multiple comparison test in the case of multiple groups. *, *P* < 0.05; **, *P* < 0.01; ***, *P* < 0.001; ****, *P* < 0.0001.

To determine how serum activin A levels in TB compare to those of other lung diseases, we investigated activin A levels in pneumonia and sarcoidosis, infectious and non-infectious diseases, respectively. Pneumonia and sarcoidosis can present with symptoms similar to TB and are relevant for differential diagnosis. No data currently exist in the public domain on serum activin A levels in these diseases; thus, we measured serum activin A levels in German pneumonia and sarcoidosis patients as well as healthy controls. Full details on the pneumonia and sarcoidosis cohorts are in Tables 3 and 4. Overall, serum activin A levels were increased in pneumonia patients compared to healthy controls (*P* < 0.001; Fig. 3A) but there was high heterogeneity among patients. There was no difference in serum activin A levels within the pneumonia group according to the CRB65 score, a clinical score used to estimate the severity of community-acquired pneumonia (32)(Fig. S3A) or age (Fig. S3B). To determine whether differences were related to pathogen type (viral versus bacterial), we compared levels in patients with proven influenza or pneumococcal pneumonia only (Fig. 3B). In both groups, activin A levels were raised compared to controls (influenza, *P* = 0.003; pneumococcal, *P* = 0.011), and there was no significant difference between the groups.

**TABLE 3:** Clinical characteristics and serum activin A levels of the pneumonia patients^*a*^

Characteristics	Pneumonia general	Pneumonia influenza	Pneumonia streptococcal	Healthy controls
	(*n* = 80)	(*n* = 25)	(*n* = 25)	(*n* = 20)
Median age, years (IQR)	65 (43–76)	69 (58–77)*	58 (41–70)	59 (56–61)
Sex, male/female, n	48/32	20/5	17/8	10/10
Physical examination findings, yes/no, n				
Fever	48/32	17/8	19/6	–
Cough	75/5	22/3	22/3	–
Production of purulent sputum	44/36	14/11	14/11	–
Pathologic lung auscultation (crackles)	51/29	18/6	18/6	–
Confusion	2/23	0/25	3/77	–
Respiratory rate >30 breaths/min	1/24	3/22	5/75	–
Systolic blood pressure <90mmHg or diastolic blood pressure<60 mmHg, y/n, n	0/25	3/22	5/75	–
CRB-65, n				
0	8	7	30	–
1	13	16	35	–
2	4	2	15	–
3	0	0	0	–
4	0	0	0	–
Median serum activin A levels (pg/mL) (IQR)	610 (461–797)^****^	477 (291–964) (368–575)^**^	522 (300–670)^**^	300 (243–350)

^*a*^ The study cohort comprised subsets of patients with pneumonia (general/all causes), pneumonia patients with proven influenza only, and pneumonia patients with proven streptococcal pneumonia only. Chest X-rays of all patients showed pulmonary infiltrates. Controls were healthy individuals from the same population (Germany). The age of influenza patients was significantly different from the age of healthy controls. Serum activin A levels were significantly increased in all pneumonia patient subsets compared to healthy controls (*P < 0.05, **P < 0.01, **** *P* < 0.0001). IQR: interquartile ratio.

**TABLE 4:** Clinical characteristics and serum activin A levels of the sarcoidosis cohort^*a*^

Characteristics	All patients	Type I	Type II	Healthy controls
	(*n* = 17)	(*n* = 9)	(*n* = 8)	(*n* = 20)
Median age, years (IQR)	46 (40–67)	52 (44–59)	41 (36–67)	59 (56–61)
Sex, male/female, n	11/6	5/4	6/2	10/10
Löfgren syndrome, yes/no, n	4/13	2/7	2/6	–
BAL AM, % (IQR)^*b*^	60 (39–83)	64 (39–87)	54 (35–68)	–
BAL L, % (IQR)^*b*^	39 (16–61)	31 (12–61)	46 (27–63)	–
BAL CD4/CD8 ratio^*b*^	4.7 (2.2–7.8)	3.3 (2.2–5.8)	6.9 (4.2–8.1)	–
Median serum activin A levels, pg/mL (IQR)	379 (351–413)^*c*^	379 (268–394)	392 (360–522)^*c*^	300 (243–350)
Median BALF activin A levels, pg/mL (IQR)	229 (175–323)	274 (195–348)	184 (122–250)	–

^*a*^ The study cohort comprised German sarcoidosis patients. Healthy German controls were the comparator group. Sarcoidosis was classified into different types according to lung pathology (Type 1: bilateral hilar adenopathy; Type II: additional involvement of pulmonary parenchyma).

^*b*^ BAL data are only available for 14/17 patients.

^*c*^ *P* < 0.01. IQR: interquartile ratio.

Serum activin A levels in German sarcoidosis patients were also compared with those of healthy controls collected during the TB and pneumonia studies. Sarcoidosis is a systemic inflammatory disorder of unknown etiology, mainly affecting the lungs, and is characterized by tissue infiltrates of lymphocytes and macrophages, and the formation of non-caseating granulomas (33). Sarcoidosis patients showed only a slight increase in serum activin A levels compared to controls (*P* < 0.01) (Fig. 2C). The individual with an outlying value of 1291 pg/mL activin A had acute renal failure in the month of blood withdrawal, with unknown etiology. There were no differences in serum or bronchoalveolar lavage fluid (BALF) activin A levels between patients with type I and type II X-ray pathology (Fig. S3C). In addition, serum activin A levels did not correlate with BALF activin A levels (Fig. S3D).

### Activin A levels in murine tuberculosis

**Fig 4:**
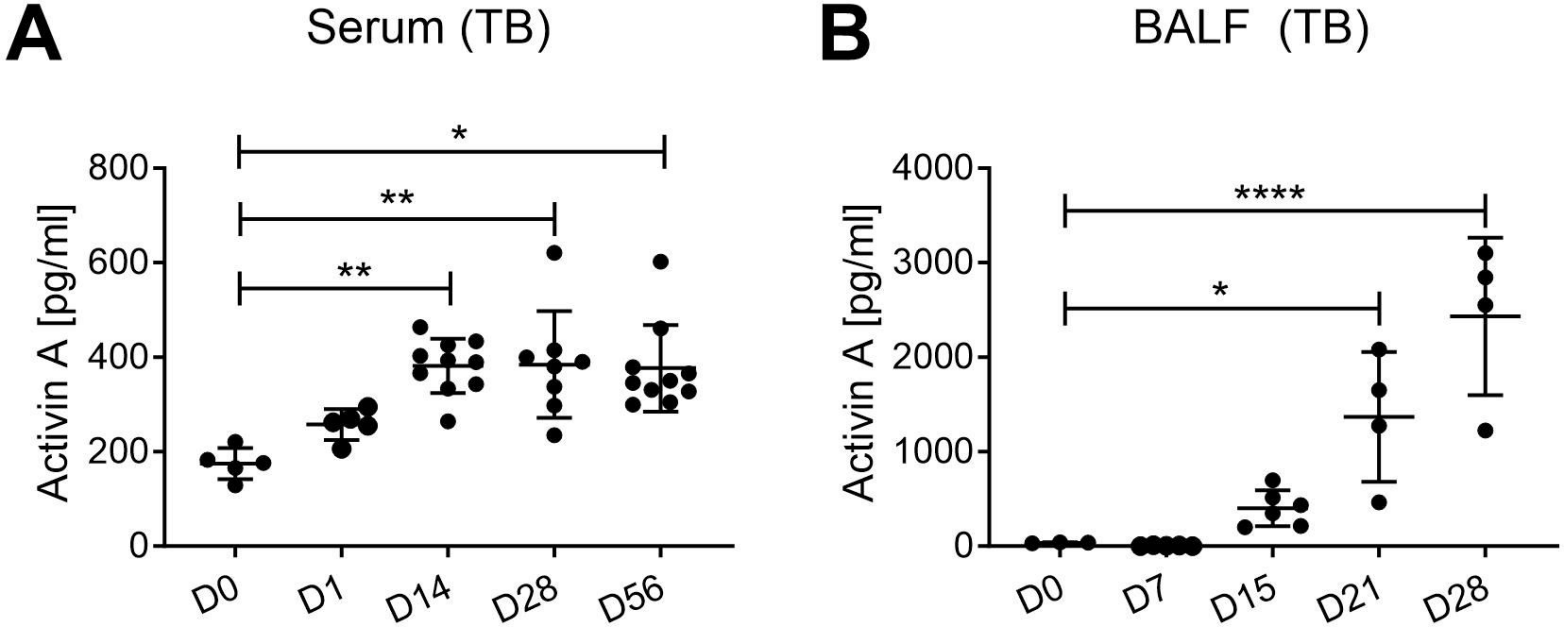
Activin A levels in bronchiolar lavage fluid (BALF) and serum in a mouse model of TB. Activin A was measured by ELISA. (**A**) Mice were infected with 100–200 CFU of *Mtb* H37Rv by aerosol inhalation and serum was collected at indicated timepoints post-infection. Control mice were naïve. D21 vs D0: *P* = 0.012; D28 vs D0: *P* < 0.0001. (**B**) BALF activin A levels in *Mtb*-infected mice and controls (see A) D14 vs D0: *P* = 0.002; D28 vs D0: *P* = 0.010; D56 vs D0: *P* = 0.015. All experiments were conducted in duplicate. Graphs show a median with an interquartile range, *n* = 3–10. Statistical significance was calculated using one-way ANOVA with Tukey’s multiple comparison test. *, *P* < 0.05; **, *P* < 0.005; ****, *P* < 0.0001.

Based on the increased levels of serum activin A in active TB disease, we embarked on investigations in mice to characterize possible functional activities of activin signaling pathways in TB. First, it was important to determine whether activin A levels were raised during murine TB, as in human disease. To investigate this, we used stored serum and BALF samples from previous studies (34). Serum activin A levels started to rise as early as day 1, but increased significantly around 2 weeks post-infection and remained elevated up until the end of the experiment (Fig. 4A). BALF activin A levels were higher than those of serum and were also raised by around 2 weeks post*-Mtb* infection (Fig. 4B). In comparison, BALF activin A levels were increased in *Streptococcus pneumoniae* (*Spn*)-infected mice quite quickly compared to naïve controls, peaking at 24 hours p.i., and then beginning to fall (Fig. S3E). Interestingly, the activin A levels mirrored the neutrophil numbers in BAL samples (Nouailles et al, unpublished data). The higher levels of activin A in BALF compared to serum in mice infected with *Mtb* suggest that activin A may be produced locally in the lungs during TB.

### Inhibition of ActRIIB signaling pathways reduces bacterial loads in a short-term model of TB in mice

**Fig 5:**
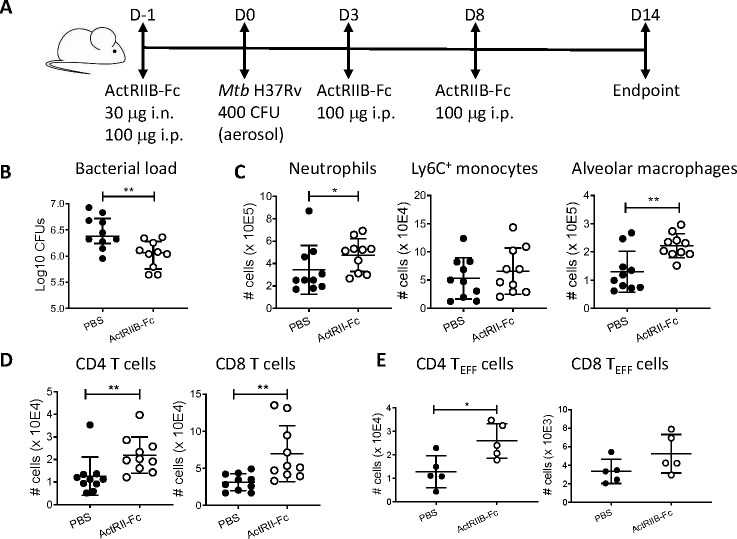
Effect of ActRIIB-Fc treatment on acute *Mtb* H37Rv infection in mice. Responses were measured in the lungs at D14 post-infection. (**A**) Experiment schedule. (**B**) Bacterial loads. (**C**) Numbers of neutrophils (*, *P* = 0.023), inflammatory (Ly6C^+^) monocytes (n.s.) and alveolar macrophages (**, *P* = 0.009). (**D**) CD4 (**, *P* = 0.002) and CD8 (**, *P* = 0.003) T-cell numbers. (**E**) Numbers of CD4 effector T cells (*, *P* = 0.032) and CD8 effector T cells (n.s.) (CD44^+^CD62L^-^). n.s. = no significant difference.

Soluble activin type IIB receptor fused to the Fc portion of human IgG1 (ActRIIB-Fc) has previously been used to ameliorate activin-induced ARDS-like pathology in mice (13). ActRIIB-Fc acts as a ligand trap for several TGF-β/activin family members with overlapping signaling pathways, including activin A, which binds ActRIIB with high affinity (14). To gain a preliminary understanding of the relevance of the activin signaling axis in TB, we investigated the effects of ActRIIB-Fc on acute experimental TB in mice. ActRIIB-Fc was administered i.n. and i.p. prior to infection, and i.p. once a week thereafter, to inhibit activin A systemically and in the lungs (Fig. 5A), which led to decreased lung bacterial loads (Fig. 5B). We analyzed lung cell populations by flow cytometry and found increased numbers of neutrophils compared to PBS-treated mice (Fig. 5C), as well as increased numbers of alveolar macrophages, which can be host cells for *Mtb* but also play a role in killing the bacilli. The number of CD4 and CD8 T cells (Fig. 5D) and CD4 effector T cells (Fig. 5E) were also increased. Therefore, not only were the activin A levels raised during *Mtb* infection but the ActRIIB signaling axis influenced cellular responses and bacterial loads.

### ActRIIB-Fc treatment increases the number of T cells with lung tissue-resident memory T (T_RM_) cell phenotype

**Fig 6:**
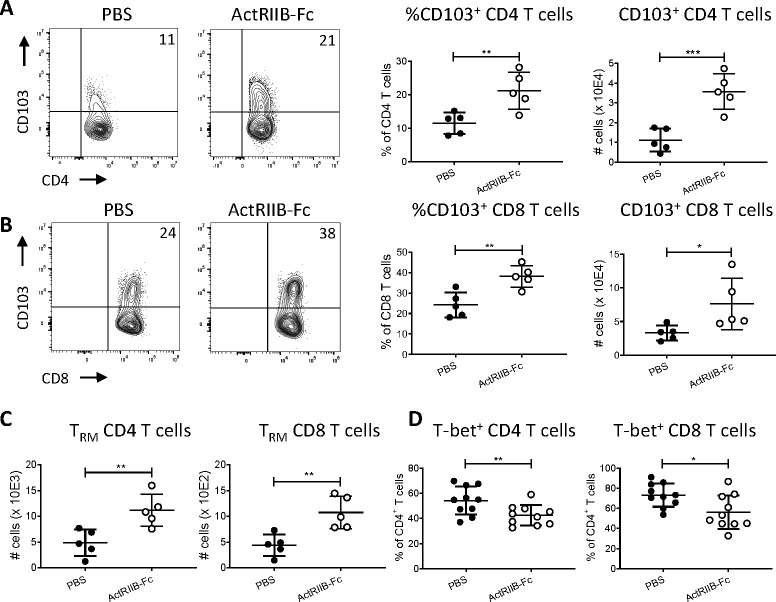
**(A**) CD103 expression on CD4 T cells. *From left to right:* Representative density plots, gated on lung CD4 T cells, depicting CD103 and CD4 expression; numbers in the upper right corner indicate % of CD103^+^ cells among CD4 T cells. Percentages of CD103^+^ cells among CD4 T cells (**, *P* = 0.009). Numbers of lung CD103^+^ CD4 T cells (***, *P* = 0.0009). (**B**) CD103 expression on CD8 T cells. *From left to right:* Representative density plots, gated on lung CD8 T cells, depicting CD103 and CD8 expression. Percentages of CD103^+^ cells among CD8 T cells (**, *P* = 0.005). Numbers of lung CD103^+^ CD8 T cells (*, *P* = 0.041). (**C**) Numbers of CD103^+^CD69^+^ CD4 T_RM_ cells (**, *P* = 0.008) and CD103^+^CD69^+^ CD8 T_RM_ cells (**, *P* = 0.008). (**D**) Percentages of T-bet^+^ CD4 (**, *P* = 0.003) and CD8 T cells (*, *P* = 0.017). All experiments were conducted in duplicate, *n* = 5 per group, and data are representative of the two experiments. Where possible, data were pooled. Graphs show a median with an interquartile range. Statistical significance was calculated using the unpaired *t*-test. *, *P* < 0.05; **, *P* < 0.005.

Because of the numerical increase in T cells after ActRIIB-Fc treatment, we analyzed phenotypes of lung T-cell populations at D14 post-infection using flow cytometry (Fig. S4A through C). The most striking finding was that blockade of ActRIIB signaling pathways in the lungs increased proportions and absolute numbers of CD103^+^ CD4, and CD103^+^ CD8 T cells (Fig. 6A and B). CD103^+^ T cells showed a spectrum of CD69 and CD44 expression (Fig. S4C). Furthermore, absolute numbers of CD4 and CD8 T_RM_ cells, defined by co-expression of CD103 and CD69, were increased (Fig. 6C), suggesting that ActRIIB-Fc signaling modulates the number of lung T_RM_ cells. We analyzed CD4 T-cell expression of transcription factors associated with Th1 (T-bet), Th17 (RORγT), and Treg (Foxp3) cells as well as with T-cell proliferation (Ki67), to determine whether sActRIIB-Fc affected T- cell differentiation and proliferation (Fig. 6D; Fig. S5A through C). T-bet expression was significantly decreased in CD4 T cells of sActRIIB-Fc treated mice compared to controls (Fig. 6D), suggesting that the ActRIIB signaling axis may affect Th1 cells, which are considered critical for TB control (35). Interestingly, the downregulation of T-bet is known to promote the expression of CD103 and T_RM_ development (36, 37). We checked T-bet expression in CD8 T cells and found that it was also decreased in ActRIIB-Fc-treated mice (Fig. 6D), concordant with the numerical increase of T_RM_ cells. Since TGF-β negatively regulates T-bet expression and directly stimulates CD103 expression *via* Smad2/3 signaling (36), we measured serum levels of TGFβ1, 2, and 3 to see whether ActRIIB-Fc treatment increased TGF-β levels but found no differences (Fig. S5D through F). By contrast, serum levels of IL-1α, IL-1β, IFN-γ, IL-17, TNF, IL-10, IL-12p40, MIP-1β, and eotaxin were all decreased in ActRIIB-Fc-treated mice (Fig. S6), indicating that ActRIIB signaling pathways affect the cytokine profile during TB, although the lower cytokine levels may also partially reflect the decreased bacterial burdens.

### ActRIIB-Fc treatment increases frequencies of lung T_RM_ cells after intranasal BCG vaccination

To see whether the effect of ActRIIB-Fc on CD103^+^ T cells was peculiar to *Mtb* infection or more general, we extended our experiments to examine responses to the vaccine strain *M. bovis* BCG in the presence of ActRIIB-Fc. T_RM_ cells are recruited to the lungs following mucosal BCG vaccination (38) and are important in controlling *Mtb* infection (38, 39). We administered ActRIIB-Fc during i.n. BCG vaccination and checked whether frequencies or numbers of T_RM_ cells were increased in the lung (Fig. S7A). Compared to PBS treatment, ActRIIB-Fc treatment increased percentages of CD103^+^ cells and T_RM_ among CD4 T cell populations (Fig. S7B and C), and caused a non-significant increase in the frequency of CD103^+^ cells and T_RM_ among CD8 T cells (Fig. S7D). Only absolute numbers of CD4 T_RM_ showed a non-significant increase after ActRIIB-Fc treatment (Fig. S7C and D). We did not pool data from replicate experiments because absolute amounts were different between individual experiments, although the trends were the same. We performed immunohistochemical staining and quantification of CD103^+^ cells in the lungs and found a non-significant increase in CD103^+^ cells in ActRIIB-Fc-treated mice (Fig. S7E and F), consistent with the flow cytometry data. Finally, we performed immunohistochemical staining of lung tissue sections for TGF-beta and did not observe significant differences in TGF-β staining between ActRIIB-Fc-treated and PBS control groups (Fig. S7G and H). The percentage of TGF-β-positive cells and the percentage of TGF-beta-positive pixels were not increased by blocking the ActRIIB signaling axis. In summary, ActRIIB-Fc increased frequencies of CD4^+^ T_RM_-like cells after intranasal BCG vaccination, and this appeared to be independent of TGF-β levels.

## DISCUSSION

This is the first study that has examined activin A levels in TB, pneumonia, and sarcoidosis. Our results demonstrate that activin A is raised during active TB and suggest that this correlates with the severity of the disease. Furthermore, experiments in mice found reduced *Mtb* burdens and changes to cellular responses during acute TB when the ActRIIB signaling axis was blocked. Our data suggest that T_RM_ cells are regulated by the ActRIIB signaling pathway.

Whole blood transcriptomic signatures, metabolites, and cytokine signatures have shown some success as host biomarkers for (i) distinguishing active TB from past or latent TB infection (LTBI), (ii) predicting progression to active TB, and (iii) monitoring treatment responses (40–43). In two independent cohorts from Gambia and Germany, activin A levels were significantly upregulated in TB patients compared to healthy controls but levels of activin A were higher in the Gambian cohort. This could be due to population differences, either environmental or genetic, or differences in cohort characteristics. In both studies, activin A levels were higher in patients with higher XRS, suggesting that activin A levels are related to the extent of the disease. Future research could evaluate whether high activin A levels post-treatment indicate ongoing infection and/or inflammation. TB patients can develop recurrent disease despite negative sputum cultures after treatment and some patients considered “cured” based on culture results still present with active lung TB lesions when checked by PET/CT scanning (44).

During the progression from LTBI to active disease, there seems to be a phase of subclinical TB, where latently infected individuals become contagious but are still healthy (45–48). In the present study, serum activin A levels showed potential for discriminating active TB patients from healthy TST^+^ individuals. Measurement of activin A levels in additional cohorts will be useful for clarifying whether activin A levels could be harnessed for triage for diagnosis of TB, that is, for identifying individuals who need a confirmatory test or are unlikely to have TB (42). Apart from activin A, we found that IP-10, IL-9, and IL-1Rα followed a similar trend being upregulated during active TB and downregulated after treatment, in agreement with previous studies (43, 49–51). Interestingly, activin A promotes Th9 differentiation in mice (20).

The gene for activin A, *INHBA*, has not featured as a leading biomarker in previous studies but expression of *INHBA* may be restricted to the site of infection and unchanged in blood cells. Furthermore, gene expression can differ from protein levels, particularly for proteins that are pre-synthesized as precursors requiring cleavage such as activin A and other TGF-beta superfamily members (52). Neutrophils are an important source of pre-formed activin A, and it has been suggested that activin A levels may be particularly raised in diseases characterized by neutrophilia (8, 13). Indeed, neutrophil levels are high in patients with active TB and decrease following successful TB treatment (53). Recently, it was shown that the activin signaling axis is upregulated during COVID-19, in which neutrophils infiltrate the lungs and correlate with severity and mortality (17, 18). IL-1α and TNF-α, which are also induced by *Mtb* infection (54, 55), induced activin A in human bronchial/tracheal smooth muscle cells and lung fibroblasts *via* the IKK/NF-κB pathway. In our cohort of pneumonia patients from the pre-COVID-19 era, serum activin A levels were heterogeneous. Studies in larger cohorts of pneumonia patients would be useful to determine whether high serum activin A levels are associated with specific factors such as disease state, dissemination to other sites, immune status, or SNPs. Larger cohorts of sarcoidosis patients could also be examined to determine whether serum activin A levels could be used in the differential diagnosis of sarcoidosis and TB where necessary.

To obtain the first idea of whether the activin signaling axis is functionally relevant in TB, we employed the ActRIIB-Fc blocker in a mouse model of acute TB infection. ActRIIB-Fc has been previously used successfully to inhibit activin A-mediated ARDS (14). Although we only looked at day 14 post*-Mtb* infection, our initial evidence shows that ActRIIB-Fc treatment decreases mycobacterial burdens and leads to increased numbers of T_RM_-like cells, characterized by the expression of CD103 and CD69 (38, 56, 57). Discrimination of vascular and parenchyma cells by intravenous injection of labeled anti-CD45 antibody prior to killing would be required to prove formally that the CD103^+^CD69^+^ T cells we observed were tissue resident (58). T cells recruited to the lung are particularly important in protective immunity to *Mtb* (38, 56, 59). CD103 (alpha E integrin; αE) expression is critical for the functional phenotype of T_RM_ cells since adhesive interactions between αE/β7 and E-cadherin allow retention of T_RM_ cells in tissue (57). Intriguingly, ActRIIB-Fc treatment led to increased numbers and proportions of CD103-expressing CD4 and CD8 T cells after both *Mtb* infection and BCG vaccination. Whether the increase in T_RM_ cell numbers due to ActRIIB-Fc treatment results in increased protection against TB remains to be investigated mechanistically. As several TGF-β superfamily family members share ActRIIB and have overlapping signaling pathways, future studies are needed that specifically target activin A to confirm and further explore its involvement in the regulation of CD103.

In summary, we demonstrate that activin A levels are increased during human TB and that the ActRIIB signaling pathway influences host responses in experimental TB in mice. This lays the groundwork for studies to investigate whether activin A or other ActRIIB ligands could be a potential target for HDT of TB (60, 61). Furthermore, activin A may be a novel biomarker for the diagnostic triage of active TB and monitoring antitubercular therapy, which should be investigated in larger cohorts from different regions.

## MATERIALS AND METHODS

Additional details can be found in the Online Supplement.

### Patients and controls

*Gambian pulmonary TB and household contact cohort* (Medical Research Council Unit The Gambia - MRCG): The demographic data of the cohort are shown in Table 1. Only HIV-negative patients were included in the cohort. The ethnicity of all the individuals was black African. In all, 30 adults with smear-positive (and subsequently culture confirmed) drug-sensitive TB were recruited and followed up to completion of treatment (standard regimen). Their TB-exposed household contacts were also recruited and analyzed for infection status by tuberculin skin testing (TST). Two independent physicians scored the X-rays according to the guidelines of the National Tuberculosis and Respiratory Disease Association of the United States (62). Blood was centrifuged in BD Vacutainer SST Tubes with Hemogard Closure (Gold) (Becton Dickinson) and serum was collected. Healthy contacts were followed up to 2 years and conversion from TST^−^ to TST^+^ or progression from TST^+^ to TB was noted. All TB cases tested (28/28) were culture negative at 6 months post-treatment (results were not available for two cases). Three individuals among the healthy household contacts were excluded due to pregnancy, which strongly raises activin A levels. One individual in the TST^+^ contact group was excluded due to diagnosis of TB on day 1 post-recruitment. Accordingly, serum activin A levels were measured in 30 TB patients at recruitment and 6 months post-treatment as well as in 29 healthy TST^−^ and 27 healthy TST^+^ contacts. Bioplex was performed on 15 samples per group.

*German pulmonary TB cohort*: Serum samples from patients with pulmonary TB (*n* = 47) and healthy controls (*n* = 27) were provided by the Research Center Borstel. The demographic data of the cohort are shown in Table 2. Since data on patient ethnicity are not usually collected in Germany due to the country’s history of racial profiling during the “Third Reich,” we are unable to provide a complete breakdown of the ethnicity of the patients. However, the majority of patients and all of the controls had Caucasian ethnicity (personal communication from Professor Christoph Lange). Only HIV-negative patients were included in the cohort. All patients had pulmonary tuberculosis and were sputum culture and sputum PCR (Xpert) positive. Of the controls, 4 were interferon-gamma release assay (IGRA) positive, 8 were IGRA negative, and 15 were not IGRA tested. The Ralph score (31) was used to quantify lung disease, calculated as the percentage of the lung being affected plus 40 points, if a cavity was present. Two independent physicians scored the X-rays.

*Pneumonia cohort*: The CAPNETZ foundation (www.capnetz.de)(63) provided serum samples of adult patients (≥18) with community-acquired pneumonia (CAP) taken within 24 hours after diagnosis. The characteristics of the cohort are shown in Table 3. Patients were identified by clinical signs (cough, purulent sputum) and a positive lung radiograph, and had not had inpatient treatment in the hospital for the previous 28 days. In all, 80 consecutive patients with pneumonia were analyzed; the causative pathogen was not recorded in all patients. Samples were collected before the COVID-19 pandemic. In addition, we analyzed serum from 25 patients considered to have proven streptococcal pneumonia and 25 patients considered to have proven influenza. The CRB65 scores in these patients were 0–2. CRB65 is a clinical score used to estimate the severity of CAP based on confusion, rate of respiration, blood pressure, and age (32). Control samples were obtained from healthy volunteers (*n* = 20). Clinical and laboratory parameters of the CAPNETZ patients are stored in an electronic database (64).

*Sarcoidosis cohort*(s): The samples were from the “Orphan Lung Biobank Freiburg” (ethics approval number 3/10). A standardized protocol was used for collecting bronchiolar lavage fluid (BALF) samples (65). Diagnosis of pulmonary sarcoidosis was based on clinical and radiological criteria with histological confirmation in lymph node or lung biopsies, as suggested by the current consensus statement of the American Thoracic Society/European Respiratory Society on sarcoidosis (66). Patients were categorized according to the Scadding radiological types of disease (67). Nine patients had been diagnosed with type I sarcoidosis (bilateral hilar adenopathy), eight with type II (additional involvement of pulmonary parenchyma), and one with type III (involvement of pulmonary parenchyma and fibrosis). The demographic data of the cohort are shown in Table 4.

### Bacterial strains

*Mycobacterium tuberculosis (Mtb*) H37Rv (American Type Culture Collection; catalog no. 27294) and BCG Danish 1331 (BCG SSI) (American Type Culture Collection; catalog no. 35733) were grown in Middlebrook 7H9 broth (BD) supplemented with albumin-dextrose-catalase enrichment (BD), 0.2% glycerol, and 0.05% Tween 80 or on Middlebrook 7H11 agar (BD) containing 10% (vol/vol) oleic acid-albumin-dextrose-catalase enrichment (BD) and 0.2% glycerol. BCG was grown to the mid-log phase, washed with phosphate-buffered saline (PBS), and stored at −80°C in PBS/10% glycerol. Prior to vaccination, BCG was thawed, washed in PBS, and prepared at a dose of 10^6^ colony forming units (CFU) in 100 µL PBS.

### Mouse models

Mice were housed in individually ventilated cages.

#### Mouse pneumonia model

Female C57BL/6J (8 – 10 weeks) were infected i.n. with 5x106 CFU of Spn serotype 2 (D39, NTCC 7466) or given PBS as a control.

#### Inhibition of ActRIIB signaling during TB or BCG vaccination

Soluble activin type IIB receptor fused to the Fc portion of human IgG1 (ActRIIB-Fc) was produced as described previously (14, 28, 68). Female C57BL/6J mice (8–10 weeks) were treated at day −2 with 30 µg ActRIIB-Fc i.n. (15 µL per nostril) and 100 µg ActRIIB-Fc i.p. (in 100 µL PBS) to inhibit activin A signaling systemically and directly in the lungs. Control mice were treated with PBS. Mice were aerosol-infected at day 0 with 400 CFU of Mtb H37Rv or vaccinated i.n. with 5 × 105 CFUs BCG. Mice were treated again on day 3 and day 8 with 100 µg ActRIIB-Fc (i.p.), or PBS as a control.

### Cytokines

Activin A was measured using the Activin A Quantikine ELISA Kit (R&D Systems). Additional cytokines and chemokines were measured using the Bio-Plex Pro TM Human Cytokine 27-Plex, Bio-Plex Pro TM Mouse Cytokine 23-Plex, and Bio-Plex Pro TM Mouse TGF-β3-plex Immunoassays (Bio-Rad).

### Mycobacterial loads

Spleens and lungs were homogenized in PBS/0.05% Tween80 (PBS-T), prepared as 10-fold serial dilutions, and plated on Middlebrook 7H11 agar containing ampicillin. CFUs were counted 3–4 weeks after incubation of the plates at 37°C.

### Flow cytometry

The left lung was cut into small pieces and digested for 1 hour at 37°C in Iscove’s Modified Dulbecco’s Medium (IMDM) (Gibco) containing 13 µg/mL DNase I (Sigma-Aldrich) and 50 U/mL collagenase IV (Sigma-Aldrich). Single-cell suspensions were prepared by passing digested tissue through 70 µM cell strainers (Corning Falcon). Isolated lung cells were stained with fluorescent antibodies (see Online Supplement) in FACS buffer (PBS/0.1% BSA) containing Fc block (anti-FcγRII/III, clone 24G2, produced in-house) and 1% rat serum and detected by flow cytometry. Intracellular staining of transcription factors was performed using the Transcription Factor Buffer set (BD Pharmingen). Cells were acquired on a Cytoflex (Beckman Coulter) (BSL3 experiments) or LSRII (BD Biosciences)(BSL2 experiments). Data were analyzed using Flowjo software (Tree Star Inc., Ashland, OR).

### Immunohistochemistry

Paraffin blocks were cut at 1 µm, and sections were mounted and dried on Superfrost Plus slides (Thermo Scientific). After dewaxing and rehydration, sections were treated with the Polyview Plus HRP Kit (Anti-mouse ENZ-KIT160-0150, anti-rabbit ENZ-KIT159-0150 Enzo, Farmingdale, N.Y. USA) following the manufacturer’s manual. CD103 (rabbit) ab224202 Abcam (Netherlands) and TGF beta-1 monoclonal antibody (mouse) MA1-21595, Invitrogen, Rockford, IL, USA, were used as primary antibodies, with an incubation time of 30 minutes at room temperature. Incubation with a blocking buffer instead of primary antibody was used as a negative control in the TGF-β staining. Sections were scanned at 40× magnification with a Zeiss Axioscan Z1 equipped with plan-apochromatic objectives. Quantification was performed with open-source software QuPath v 0.3.2 (69).

### Statistics

GraphPad Prism 8 was used for statistical analysis. For comparing two groups, the Mann-Whitney test or unpaired one-tailed *t-*test was used, as indicated in figure legends. For comparing multiple groups, the Kruskal-Wallis test with Dunn’s multiple comparison test or the one-way ANOVA with Tukey’s multiple comparisons test was used, as indicated in figure legends. Linear regression was used for correlation calculations. The logistic regression model was used to determine the ability of activin A levels, IP-10 levels or activin A and IP-10 levels in combination with discriminate between groups. For the obtained models, the receiver operating characteristics (ROC) were calculated together with the area under the curve (AUC). The non-parametric DeLongs algorithm was used to assess statistical differences between ROCs (70, 71), using the pROC and ggplot2 package in R 4.2.2. For binary variable dependency, the Jonckheere-Terpstra trend test was used; for other cases, the Spearman rank correlation was calculated, accompanied by linear regression. *P* < 0.05 was considered significant.

### Study approvals

For the clinical samples, all subjects, or their legal representatives, gave written informed consent in accordance with the Declaration of Helsinki, and study protocols were approved by local ethics committees. The tuberculosis study was approved by the MRCG/Gambian government joint ethics committee (reference number SCC1333). The pneumonia study was approved by central and local ethics committees (Ethics Committee of the Hannover Medical School (MHH); registration number: 301–2008). The sarcoidosis samples were taken from the “Orphan Lung Biobank Freiburg” (ethics approval number 3/10). Samples from the Research Center Borstel were collected for a tuberculosis biomarker project (Ethics Committee of the University of Lübeck, AZ 12–233, Germany).

All mouse experiments were ethically approved by the State Ofﬁce for Health and Social Services, Berlin, Germany (project numbers G0266/11, G0139/14, G0376/13, and G0250/16). Mice were handled in accordance with the European directive 2010/63/EU on the Care, Welfare and Treatment of Animals. All efforts were taken to minimize their discomfort.

## SUPPLEMENTAL MATERIAL

Supplemental Material mbio.03408-23-s0001.docx Study group members and supplemental text, tables, and figures.

ASM does not own the copyrights to Supplemental Material that may be linked to, or accessed through, an article. The authors have granted ASM a non-exclusive, world-wide license to publish the Supplemental Material files. Please contact the corresponding author directly for reuse.
