# Supplementary material for: Activin A levels are raised during human tuberculosis and blockade of the activin signaling axis influences murine responses to M. tuberculosis infection
Source: mBio. 2024 Feb 20;15(3):e03408-23. doi: 10.1128/mbio.03408-23 (PMC10936190; doi:10.1128/mbio.03408-23)
Supplement: Supplemental Material — Study group members and supplemental text, tables, and figures. [file mbio.03408-23-s0001.docx]

**ONLINE SUPPLEMENT**

**Activin A levels are raised during human tuberculosis and blockade of the activin signaling axis influences murine responses to *M. tuberculosis* infection**

Natalie E. Nieuwenhuizen^1,2^, Geraldine Nouailles^3^, Jayne S. Sutherland^4^, Joanna Zyla^5^, Arja H. Pasternack^6^, Jan Heyckendorf^7^, Björn C. Frye^8^, Kerstin Höhne^8^, Ulrike Zedler^1^, Silke Bandermann^1^, Ulrike Abu Abed^9^, Volker Brinkmann^9^, Birgitt Gutbier^3^, Martin Witzenrath^3,10,11^, Norbert Suttorp^3,10,11^, Gernot Zissel^8^, Christoph Lange^12^, Olli Ritvos^6^, Stefan HE Kaufmann^1,13,14^, the CAPNETZ Study group^10^ and the DZIF TB study group^15^

1 Department of Immunology, Max Planck Institute for Infection Biology, Chariteplatz 1, 10117, Berlin, Germany

2 Institute for Hygiene and Microbiology, University of Würzburg, Josef-Schneider-Str.2, 97080 Würzburg, Germany

3 Department of Infectious Disease and Respiratory Medicine, Charité – Universitätsmedizin Berlin, corporate member of Freie Universität Berlin, Humboldt-Universität zu Berlin, and Berlin Institute of Health, Charitéplatz 1, 10117 Berlin, Germany

4 Medical Research Council Unit The Gambia (MRCG), at the London School of Hygiene and Tropical Medicine, Fajara, The Gambia.

5 Department of Data Science and Engineering, Silesian University of Technology, Gliwice, Poland

6 Department of Physiology, Faculty of Medicine, University of Helsinki, Helsinki, Finland

7 Department of Medicine I, University Hospital Schleswig-Holstein, Kiel, Germany

8 Department of Pneumology, Medical Center – University of Freiburg, Faculty of Medicine, Hugstetter. Str. 55, 79106 Freiburg, Germany

9 Microscopy Core Facility, Max Planck Institute for Infection Biology, Chariteplatz 1, 10117, Berlin, Germany

10 CAPNETZ STIFTUNG, Hannover, Germany. Members are listed in the supplemental material.

11 German Center for Lung Research (DZL)

12 ^a^Division of Clinical Infectious Diseases, Research Center Borstel, Borstel, Germany. ^b^German Center for Infection Research (DZIF), Partner Site Hamburg-Lübeck-Borstel-Riems, Borstel, Germany; ^c^Respiratory Medicine and International Health, University of Lübeck, Lübeck, Germany; ^d^Baylor College of Medicine and Texas Children´s Hospital, Global TB Program, Houston, TX, USA;

13 ^a^Max Planck Institute for Multidisciplinary Sciences, Emeritus Group Systems Immunology, Am Faßberg, 1137077, Göttingen, Germany

14 Hagler Institute for Advanced Study, Texas A&M University, College Station, TX, United States.

15 German Center for Infection Research (DZIF), Braunschweig, Germany. Members are listed in the supplemental material.

**Corresponding author:** Natalie E. Nieuwenhuizen, Institute for Hygiene and Microbiology, Julius-Maximilians-Universität Würzburg, Josef-Schneider-Str.2, 97080 Würzburg, Germany. Email: [natalie.nieuwenhuizen@uni-wuerzburg.de](mailto:natalie.nieuwenhuizen@uni-wuerzburg.de)

**Members of the CAPNETZ study group:** M. Dreher, C. Cornelissen (Department of Pneumology and Intensive Care Medicine, University Hospital Aachen); W. Knüppel (Department of Internal Medicine, Bad Arolsen Hospital); D. Stolz (Department of Pneumology, University Hospital Basel, Switzerland); W. Bauer (Central Emergency Admission / Medical Admission Ward, Charité-Universitätsmedizin Berlin); N. Suttorp, A. Mikolajewska, M. Witzenrath (Medical Department, Division of Infectiology and Pneumonology, Charité-Universitätsmedizin Berlin); S. Gläser, D. Thiemig (Department of Internal Medicine - Pneumology and Infectiology, Vivantes Hospital Neukölln, Berlin-Neukölln); C. Boesecke (Medical Clinic and Polyclinic I – General Internal Medicine, University Hospital Bonn); M. Prediger, S. Schmager (III. Medical Clinic, Carl-Thiem Hospital Cottbus); B. Schaaf, J. Kremling, D. Nickoleit-Bitzenberger (Pneumology, Infectiology and Internal Intensive Care Medicine, Medical Clinic Nord, Dortmund); M. Kolditz, B. Schulte-Hubbert, S. Langner (Medical Clinic I Department of Pneumology, University Hospital Dresden); G. Rohde (Medical Clinic I - Pneumology/Allergology, University Hospital Frankfurt), M. Panning (Institute of Virology, University Hospital Freiburg); C. Neurohr (Department of Pneumology and Respiratory Medicine, Clinic Schillerhöhe, Gerlingen); T. Welte, I. Pink (Department of Pneumology, Hannover Medical School, Hannover); T. Fühner, M. van’t Klooster (Department of Pneumology, Intensive Care and Sleep Medicine, Siloah Hospital, Hannover), G. Barten, W. Kröner, F. Eberherdt, O. Unruh, N. Adaskina (CAPNETZ Office, Hannover); T. Illig, N. Klopp (Hannover Unified Biobank, Hannover Medical School); M. Pletz (Institute for Infection Medicine and Hospital Hygiene (IIMK), University Hospital Jena); D. Drömann, P. Parschke, K. Franzen (Medical Clinic III, Pneumology/Infectiology, University Medical Center Schleswig-Holstein, Lübeck); J. Rupp, N. Käding, F. Waldeck (Department of Infectious Diseases and Microbiology, University Hospital Schleswig-Holstein, Lübeck); C. Spinner (Department of Internal Medicine II, University Hospital rechts der Isar, Technical University of Munich, School of Medicine); D. Heigener, I. Hering (Department of Pneumology, Agaplesion Diakonieklinikum Rotenburg); W. Albrich, M. Seneghini, F. Rassouli, S. Baldesberger (Department of Infectiology and Hospital Hygiene, Kantonsspital St. Gallen, Switzerland); S. Stenger (Institute for Medical Microbiology and Hygiene, University Hospital Ulm), M. Wallner (2mt Software, Ulm); H. Burgmann, L. Traby, L. Schubert, R. Chen (University Clinic for Internal Medicine I, Medical University of Vienna); and all study nurses.

**Members of the DZIF-TB cohort study group:** Jan Heyckendorf^1^, Maja Reimann^2,3,4^, Korkut Avsar^5^, Andrew DiNardo^6^, Gunar Günther^7,8^, Michael Hoelscher^9,10^, Elmira Ibraim^11^, Barbara Kalsdorf^2,3,4^, Stefan H.E. Kaufmann^12,13,14^, Irina Kontsevaya^1,2,3,15^, Frank van Leth^16^, Anna Maria Mandalakas^2,3,17^, Florian P. Maurer^18,19^, Marius Müller^20^, Dörte Nitschkowski^21,22^, Ioana D. Olaru^23,24^, Cristina Popa^11^, Andrea Rachow^9,10^, Thierry Rolling^25,26^, Jan Rybniker^27,28,29^, Helmut J. F. Salzer^30^, Patricia Sanchez-Carballo^2,3,4^, Maren Schuhmann^31^, Dagmar Schaub^2,3,4^, Victor Spinu^12^, Isabelle Suárez^27^, Elena Terhalle^2,3,4^, Markus Unnewehr^32,33^, January Weiner 3^rd 34^, Torsten Goldmann^21,22^, Christoph Lange^2,3,4,17^

^1^ Department of Medicine I, University Hospital Schleswig-Holstein, Kiel, Germany; ^2^Division of Clinical Infectious Diseases, Research Center Borstel, Borstel, Germany; ^3^German Center for Infection Research (DZIF), Germany; ^4^International Health/Infectious Diseases, University of Lübeck, Lübeck, Germany; ^5^Asklepios Fachkliniken München-Gauting, Munich, Germany; ^6^The Global Tuberculosis Program, Texas Children’s Hospital, Immigrant and Global Health, Department of Pediatrics, Baylor College of Medicine, Houston, USA; ^7^Department of Medicine, University of Namibia School of Medicine, Windhoek, Namibia; ^8^Inselspital Bern, Department of Pulmonology, Bern, Switzerland; ^9^Division of Infectious Diseases and Tropical Medicine, University Hospital, LMU Munich, Munich, Germany; ^10^German Center for Infection Research (DZIF), partner site Munich, Germany; ^11^Institutul de Pneumoftiziologie "Marius Nasta", MDR-TB Research Department, Bucharest, Romania; ^12^Max Planck Institute for Infection Biology, Berlin, Germany; ^13^Max Planck Institute for Biophysical Chemistry, Göttingen, Germany; ^14^Hagler Institute for Advanced Study, Texas A&M University, College Station, USA; ^15^Department of Infectious Disease, Faculty of Medicine, Imperial College London, London, UK; Department of Health Sciences, VU University; ^16^Amsterdam Public Health Research Institute, Amsterdam, The Netherlands; ^17^The Global Tuberculosis Program, Texas Children’s Hospital, Immigrant and Global Health, Department of Pediatrics, Baylor College of Medicine, Houston, USA; ^18^ National and WHO Supranational Reference Center for Mycobacteria, Research Center Borstel, Borstel, Germany^; 19^Institute of Medical Microbiology, Virology and Hygiene, University Medical Center Hamburg-Eppendorf, Hamburg, Germany; ^20^Sankt Katharinen-Krankenhaus, Frankfurt, Germany; ^21^Pathology of the Universal Medical Center Schleswig-Holstein (UKSH) and the Research Center Borstel, Campus Borstel, Airway Research Center North (ARCN); ^22^German Center for Lung Research (DZL), Germany; ^23^London School of Hygiene and Tropical Medicine, London, United Kingdom; ^24^Biomedical Research and Training Institute, Harare, Zimbabwe; ^25^Division of Infectious Diseases, I. Department of Internal Medicine, German Center for Infection Research (DZIF); University Medical Centre Hamburg-Eppendorf, Hamburg, Germany, ^26^Department of Clinical Immunology of Infectious Diseases, Bernhard-Nocht-Institute for Tropical Medicine, Hamburg, Germany; ^27^Department I of Internal Medicine, Division of Infectious Diseases, University of Cologne, Cologne, Germany; ^28^German Center for Infection Research (DZIF), Partner Site Bonn-Cologne, Cologne, Germany; ^29^Center for Molecular Medicine Cologne, University of Cologne, Cologne, Germany; ^30^Department of Pulmonology, Kepler University Hospital, Linz, Austria; ^31^Universitäts Thoraxklinik-Heidelberg, Heidelberg, Germany; ^32^Department of Respiratory Medicine and Infectious Diseases, St. Barbara-Klinik, Hamm, Germany; ^33^University of Witten-Herdecke, Witten, Germany; ^34^Berlin Institute of HealthCUBI (Core Unit Bioinformatics), Berlin, Germany

**EXTENDED METHODS**

**Patients and controls**

*Gambian pulmonary TB and household contact cohort* (Medical Research Council Unit The Gambia - MRCG): The demographic data of the cohort is in Table 1. The ethnicity of all the individuals was black African. Thirty adults with smear positive (and subsequently culture-confirmed drug-sensitive tuberculosis (TB) were recruited following written informed consent and followed up to completion of treatment (standard regimen). Their TB-exposed household contacts were also recruited and analyzed for infection status by tuberculin skin-testing (TST). Only HIV-negative adults were included in our cohort. Two independent physicians scored the X-rays according to the guidelines of the National Tuberculosis and Respiratory Disease Association of the U.S.A. (1). Blood was collected into BD Vacutainer SST Tubes with Hemogard Closure (Gold) (Becton Dickinson), centrifuged and serum stored at -80^o^C until processing. Healthy contacts were followed up to 2 years and conversion from TST- to TST+ or progression from TST+ to TB was noted. All TB cases tested (28/28) were culture-negative at 6 months post treatment (results were not available for 2 cases).

Three individuals among the healthy household contacts were excluded due to pregnancy, which is known to strongly raise activin A levels. One individual in the TST+ contact group was excluded due to diagnosis at day 1 post recruitment with TB based on clinical symptoms (productive cough for over 2 weeks, chest and side pains), and started drug treatment. This individual had minimal signs of disease on the X-rays, and did not have raised serum activin A (260 pg/ml). Accordingly, serum activin A levels were measured in 30 TB patients at recruitment and 6 months post treatment as well as in 29 healthy TST- and 27 healthy TST+ contacts. Bioplex was performed on 15 samples per group.

**German pulmonary TB cohort**

Serum samples from patients with pulmonary TB (n = 47) and healthy controls (n = 27) were provided by the Research Center Borstel. Only HIV negative patients were included in the cohort. All patients had pulmonary tuberculosis as the main manifestation of disease, and were sputum culture and sputum PCR (Xpert) positive. Since data on patient ethnicity are not usually collected in Germany due to the country’s history of racial profiling during the "Third Reich", we are unable to provide a complete breakdown of ethnicity of the patients. However, the majority of patients and all of the controls had Caucasian ethnicity (personal communication from Professor Christoph Lange). Of the controls, 4 were interferon-gamma release assay (IGRA) positive, 8 were IGRA negative, and 15 were not IGRA tested. The Ralph score (2) was used to quantify lung disease, calculated as the percentage of lung being affected plus 40 points, if a cavity was present. Two independent physicians scored the X-rays. The demographic data of the cohort is given in Table 2.

**Pneumonia cohort**

The CAPNETZ foundation (www.capnetz.de)(3) provided serum samples of adult patients (≥18) with community-acquired pneumonia (CAP) taken within 24 hours after diagnosis. Patients were identified by clinical signs (cough, purulent sputum) and a positive lung radiograph, and had not had inpatient treatment in the hospital for the previous 28 days. Eighty consecutive patients with pneumonia were analysed, with the causes of pneumonia including *Streptococcus* spp., *Klebsiella* spp. and *Haemophilus* spp. The causative pathogen was not recorded in all patients. Samples were collected before the covid pandemic. In addition, we analysed serum from 25 patients considered to have proven streptococcal pneumonia (main pathogen *Streptococcus* *pneumoniae*; *Pasteurella multocida* detected in one patient, *Aspergillus* detected in one patient) and 25 patients considered to have proven influenza (main pathogen Influenza virus A (n = 21) or Influenza virus B (n = 4); *Staphylococcus aureus* and *Escherichia coli* each detected in one patient, *Candida albicans* detected in two patients). The CRB65 scores in these patients were 0-2. CRB65 is a clinical score used to estimate the severity of CAP based on confusion, rate of respiration, blood pressure and age (4). The score is generated by giving one point each for: Confusion, Rate of respiration ≥30, Blood pressure: systolic BP <90 mmHg or diastolic BP ≤60 mmHg, and age: 65 and over. Control samples were obtained from healthy volunteers (n = 20). Clinical and laboratory parameters, microbiology of respiratory pathogens, comorbidities, length of hospital stay and mortality of the CAPNETZ patients are stored in an electronic database (5). The characteristics of the CAPNETZ patient cohort used in the present study are in Table 3.

**Sarcoidosis cohort**

The samples were from the “Orphan Lung Biobank Freiburg” (ethics approval number 3/10). All patients gave their written informed consent. A standardized protocol was used for collecting bronchiolar lavage fluid (BALF) samples (6). Briefly, 300ml of pre-warmed isotonic NaCl solution was instilled in 20ml portions with immediate manual suction. Diagnosis of pulmonary sarcoidosis was based on clinical and radiological criteria with histological confirmation in lymph node or lung biopsies, as suggested by the current consensus statement of the American Thoracic Society/European Respiratory Society on sarcoidosis (7). Patients were categorized according to the Scadding radiological types of disease (8). 9 patients had been diagnosed with type I sarcoidosis (bilateral hilar adenopathy), 8 with type II (additional involvement of pulmonary parenchyma), and one with type III (involvement of pulmonary parenchyma and fibrosis)]. The demographic data of the cohort is in Table 4.

**Mouse pneumonia model**

*Streptococcus pneumonia (Spn)* serotype 2 (D39, NTCC 7466) was grown for 8 h on BD™ Columbia Agar with 5% Sheep Blood at 37°C, 5 % CO2. Single colonies were transferred to Todd Hewitt Broth medium supplemented with 0.5% yeast and 10% heat-inactivated fetal calf serum (FCS) and grown to mid-log phase. Bacteria were collected by centrifugation and washed with 1x PBS.

Under anesthesia, female C57BL/6J (8 – 10 weeks) were infected i.n. with 5x10^6^ CFU of *Spn* serotype 2 (D39, NTCC 7466) in 20 µl 1x PBS. Control mice received 20 µl sterile 1x PBS i.n. At the corresponding time points of analysis, mice were anaesthetized by intraperitoneal administration of ketamine and xylazine. Upon loss of pedal withdrawal reflexes, blood was drawn from the vena cava and the mice were sacrificed by exsanguination. Airways were washed twice with 800 μl PBS. The resulting cell suspensions were centrifuged and the supernatants (BALF) were stored at − 80 °C until ELISAs were performed.

**Inhibition of ActRIIB signaling during TB or BCG vaccination:** Soluble activin type IIB receptor fused to the Fc portion of human IgG1 (ActRIIB-Fc) was produced as described previously (9-11). ActRIIB-Fc is well tolerated and exhibits low clearance and a relatively long serum half-life (3-7 days) after a single dose in mice (12). Previous studies demonstrated that doses of 5mg/kg effectively inhibit activin A signalling in mice, with no increase in effect seen at 10mg/kg (10). Based on preliminary work, we added an intranasal dose to previously published protocols in order to improve neutralization of activin A in the lungs. Female C57BL/6J mice (8-10 weeks) were treated at day -2 with 30 μg ActRIIB-Fc i.n. (15 μl per nostril) and 100 μg ActRIIB-Fc i.p. (in 100 μl PBS) to inhibit activin A signalling systemically and directly in the lungs. Control mice were treated with PBS. Mice were aerosol-infected at day 0 with 400 CFU of Mtb H37Rv or vaccinated i.n. with 5x105 CFUs BCG. Mice were treated again at day 3 and day 8 with 100 μg ActRIIB-Fc (i.p.), or PBS as a control.

**Antibodies used for flow cytometry**

Anti-CD45 Brilliant Violet 785 (Biolegend, clone 30-F11), anti-CD3 Alexa 700 (BioLegend, clone 17A2), anti-CD4 V500 (BD Biosciences, clone RM4-5), anti-CD8 PerCP (Biolegend, clone 53-6.7), anti-CD62L APC (BD Biosciences, clone MEL-14), anti-CD44 Paciﬁc Blue (clone IM7, grown in-house), anti-CD103 PE (Biolegend, clone 2E7), anti-CD69 PE-Cy7 (Biolegend, clone H1.2F3), anti-CD25 PE/Dazzle 594 (Biolegend, clone 3C7), anti-CD45 Alexa 700 (Biolegend, clone 30-F11), anti-TCR-β Brilliant Violet 605 (BD Bioscience, clone H57-597), anti-CD4 PEC7 (Biolegend, clone RM4-5), anti-CD44 BV510 (BD Bioscience, clone I7), anti-T-bet PE (Biolegend, clone 4B10), anti-CD45 FITC (Biolegend, clone 30-F11), anti-CD45 785 (Biolegend, clone 30-F11), anti-CD11c APC (Biolegend, clone N418), anti-Siglec F PE (BD Biosciences,clone E50-2440) , anti-CD11b V500 (BD Biosciences, clone M1/70), anti-MHCII Alexa 700 (Biolegend, clone M5/114.15.2, anti-Ly6C Pacific blue (Biolegend, clone HK1.4), anti-Ly6G PerCP (Biolegend, clone 1A8).

**SUPPLEMENTARY TABLES**

**Table S1:** Ability of activin A and/or IP-10 to discriminate TB patients from controls

| **GAMIBIA - AUC with 95% CI** | | | |
| --- | --- | --- | --- |
|  | **Activin A** | **IP-10** | **Both** |
| **TST^+^** | 0.85 [0.71 - 1.00] *** | 0.85 [0.70 -1.00] *** | 0.93 [0.84 - 1.00] **** |
| **TST^-^** | 0.98 [0.95 -1.00] **** | 0.86 [0.70 - 1.00] *** | 0.99 [0.98 - 1.00] **** |
| **GERMAN - AUC with 95% CI** | | | |
|  | **Activin A** | **IP-10** | **Both** |
| **all TB** | 0.76 [0.65 - 0.87] *** | 0.80 [0.69 - 0.91] **** | 0.82 [0.72 - 0.91] **** |
| **GERMAN - BY TB type - AUC with 95% CI** | | | |
|  | **MDR** | **XDR** | **Drug sensitive** |
| **IP-10** | 0.78 [0.64 - 0.92]*** | 0.83 [0.68 - 0.97]** | 0.82 [0.68 - 0.96]*** |
| **Activin A** | 0.80 [0.67 - 0.94]*** | 0.84 [0.71 - 0.98]*** | 0.66 [0.46 - 0.86] ^NS^ |
| **Both** | 0.81 [0.68 - 0.94]**** | 0.84 [0.69 - 0.99]*** | 0.82 [0.68 - 0.96]**** |

Serum activin A levels were measured by ELISA; IP-10 levels were measured using the Bio-Plex Pro TM Human Cytokine 27-Plex immunoassay in the Gambian patients and by IP-10 ELISA in the German patients. TST^+^ and TST^-^: tuberculin skin test (TST) positive and negative healthy contacts of TB patients. MDR: multi-drug resistant. XDR: extensively drug resistant. The logistic regression model was used to determine the ability of activin A levels, IP-10 levels or activin A and IP-10 levels in combination to discriminate between groups. The receiver operating characteristics (ROC) were calculated for the obtained models together with the area under the curve (AUC) with its 95% confidence interval (CI). The non-parametric DeLongs algorithm was used to assess statistical differences between different ROCs. NS, not significantly different; **, P<0.01; ***, P<0.001; ****, P<0.0001.

**Table S2:** Comparison of the Gambian and German TB patients

|  | | **Gambian**  **cohort** | **German cohort** |
| --- | --- | --- | --- |
| **Characteristic** | | (n = 30) | (n = 47) |
| Median age, yrs. (IQR) | 27 (21-34) | | 42 (28-47)*** |
| Sex |  | |  |
| male/female, n | 15/15 | | 31/16 |
| Drug resistance, % |  | |  |
| Drug sensitive TB | 100% | | 38.3% |
| MDR TB | 0% | | 40.4% |
| XDR TB | 0% | | 21.3% |
| Smear grade (BC), n |  | |  |
| - | 0 | | 14 |
| -/+ | 0 | | 8 |
| + | 6 | | 7 |
| ++ | 9 | | 7 |
| +++ | 15 | | 11 |
| X ray score, n |  | |  |
| 0 | 0 | | 0 |
| 1 | 1 | | 11 |
| 2 | 13 | | 10 |
| 3 | 15 | | 26 |
| Median Activin A levels, pg/ml (IQR) | 811 (644-1101) | | 425 (302-629)**** |

Serum samples and clinical data were collected from pulmonary TB patients in Gambia and Germany. MDR = multi-drug resistant; XDR = extensively drug resistant. X-ray scores were not available for one individual in the Gambian cohort. X ray score: 0, normal; 1, minimal disease; 2, moderate disease; 3, advanced disease. Ralph scores were recorded for the German TB patients (% lung affected plus 40 points if cavities were present). Ralph scores were converted to a 1-3 scale as follows for comparison with the Gambian cohort scores: >20: minimal disease; 21-59: moderate disease; >60: advanced disease. SG: -, negative; +/-, scanty (1–9 AFB in 100 fields); +, 10-99 AFB in 100 fields; ++, 1-10 AFB per field; +++, more than 10 AFB per field. Median patient ages and serum activin A levels were significantly different in the two cohorts (****P*<0.001, **** *P*<0.0001). IQR: interquartile ratio.

**SUPPLEMENTARY FIGURES**


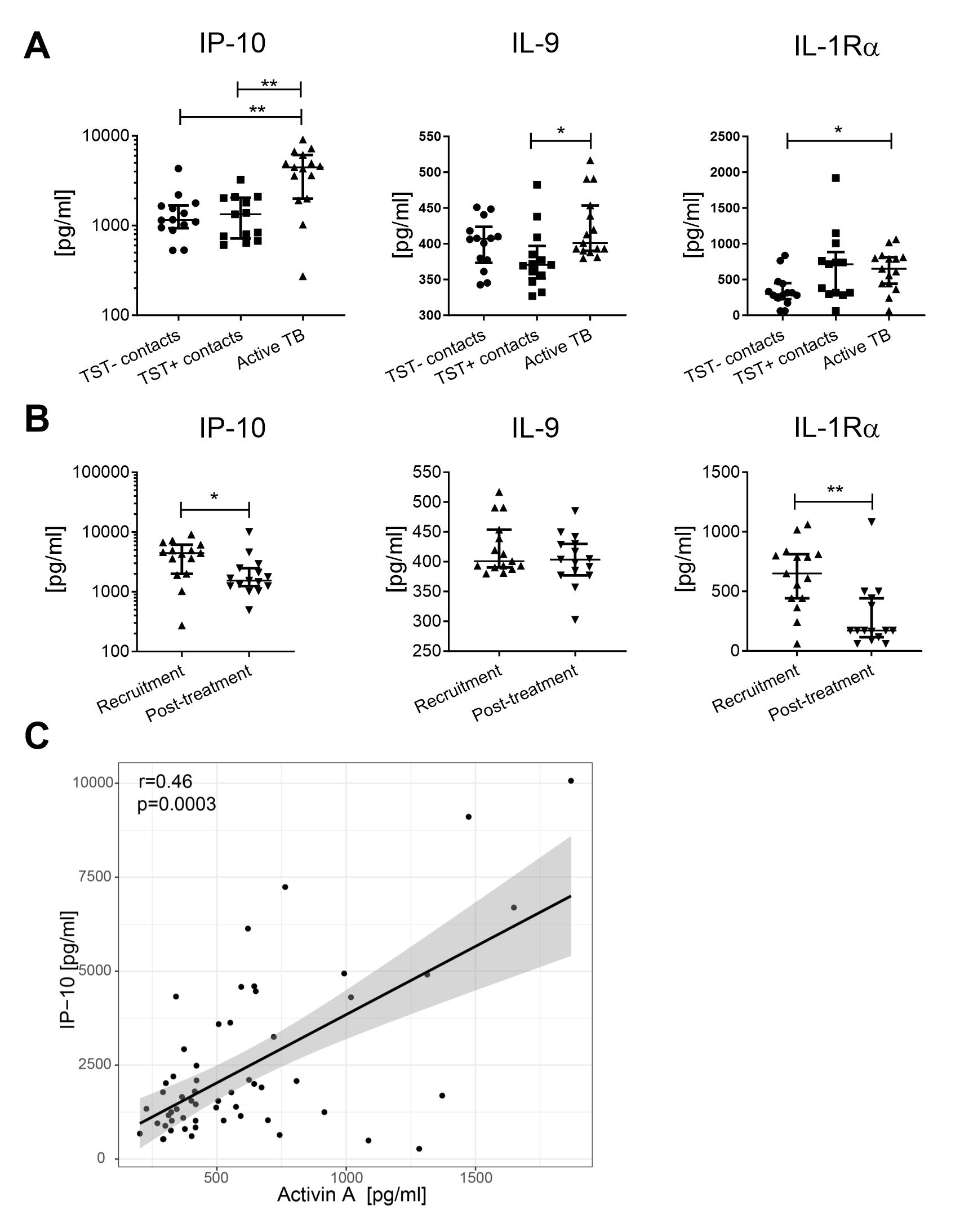


**Fig. S1.** Serum IP-10 is increased in active TB compared to TST^-^ and TST^+^ contacts. Analysis of 27 cytokines and chemokines in serum of active TB patients and TST^-^ and TST^+^ contacts was performed using the Bio-Plex Pro ^TM^ Human Cytokine 27-Plex immunoassay. Only cytokines with significant differences between groups are shown. A) Serum IP-10, IL-9 and IL-1Rα levels in patients with active TB and in TST^-^ and TST^+^ contacts. (IL-9: *, P = 0.011; IL-1Rα: *, P = 0.029) Statistical significance was calculated using the Kruskal-Wallis test with Dunn’s multiple comparison test. B) Serum IP-10, IL-9 and IL-1Rα levels in TB patients at recruitment and 6 months post treatment (IP-10: *, *P* = 0.030). Statistical significance was calculated using the Wilcoxon matched pairs signed rank test. *, *P*<0.05; **, *P*<0.01. C) Correlation between activin A and IP-10 levels. Pearson’s correlation with t-test was calculated and results are accompanied by linear regression.


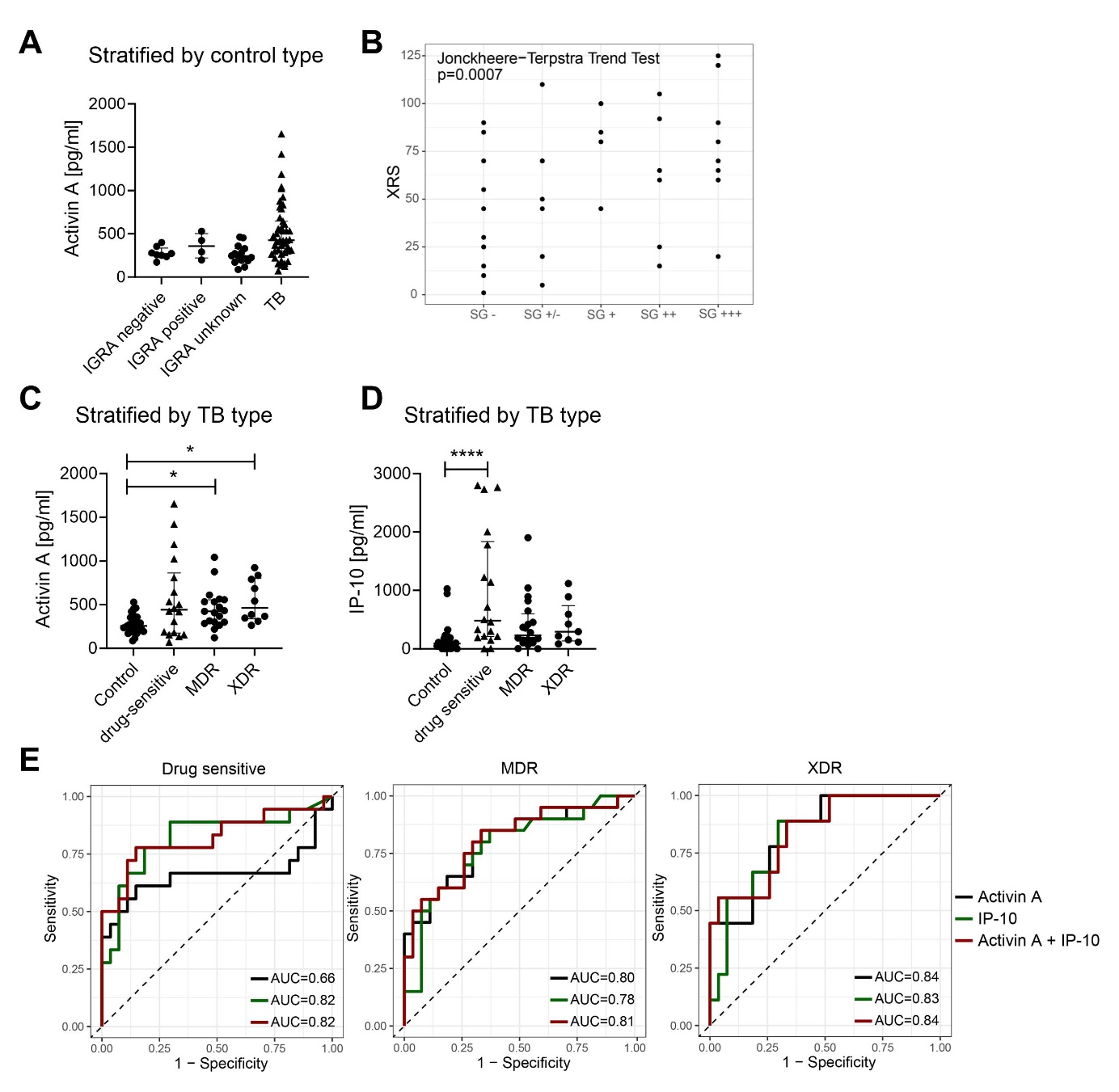


**Fig. S2.** A) Serum activin A levels in patients with active TB (n = 47)(German cohort), with healthy controls stratified into IGRA negative (n = 8), positive (n = 4) and IGRA status unknown (n = 15) groups. Activin A was measured by ELISA. B) Jonckheere-Terpstra trend test to determine whether there was a trend to increased X-ray scores (XRS) with increased smear grade level (SG) in the German TB cohort. XRS was generated using the Ralph method. Smears were classified as SG: -, negative; +/-, scanty (1–9 AFB in 100 fields); +, 10-99 AFB in 100 fields; ++, 1-10 AFB per field; +++, more than 10 AFB per field. C) Serum activin A levels in patients with active TB (German cohort), stratified according to drug-susceptiblity of the casuative *M. tuberculosis* strain: drug sensitive (n = 18), multi-drug resistant (MDR)(n = 19, *P* = 0.021) or extensively drug resistant (XDR)(n = 10, *P* = 0.013). D) Serum IP-10 levels in patients with active TB (German cohort), stratified according to drug-susceptiblity of the casuative *M. tuberculosis* strain. E) ROC curves showing the ability of activin A, IP-10 and a combination of activin A and IP-10 to discriminate drug sensitive, MDR and XDR TB patients from healthy controls.


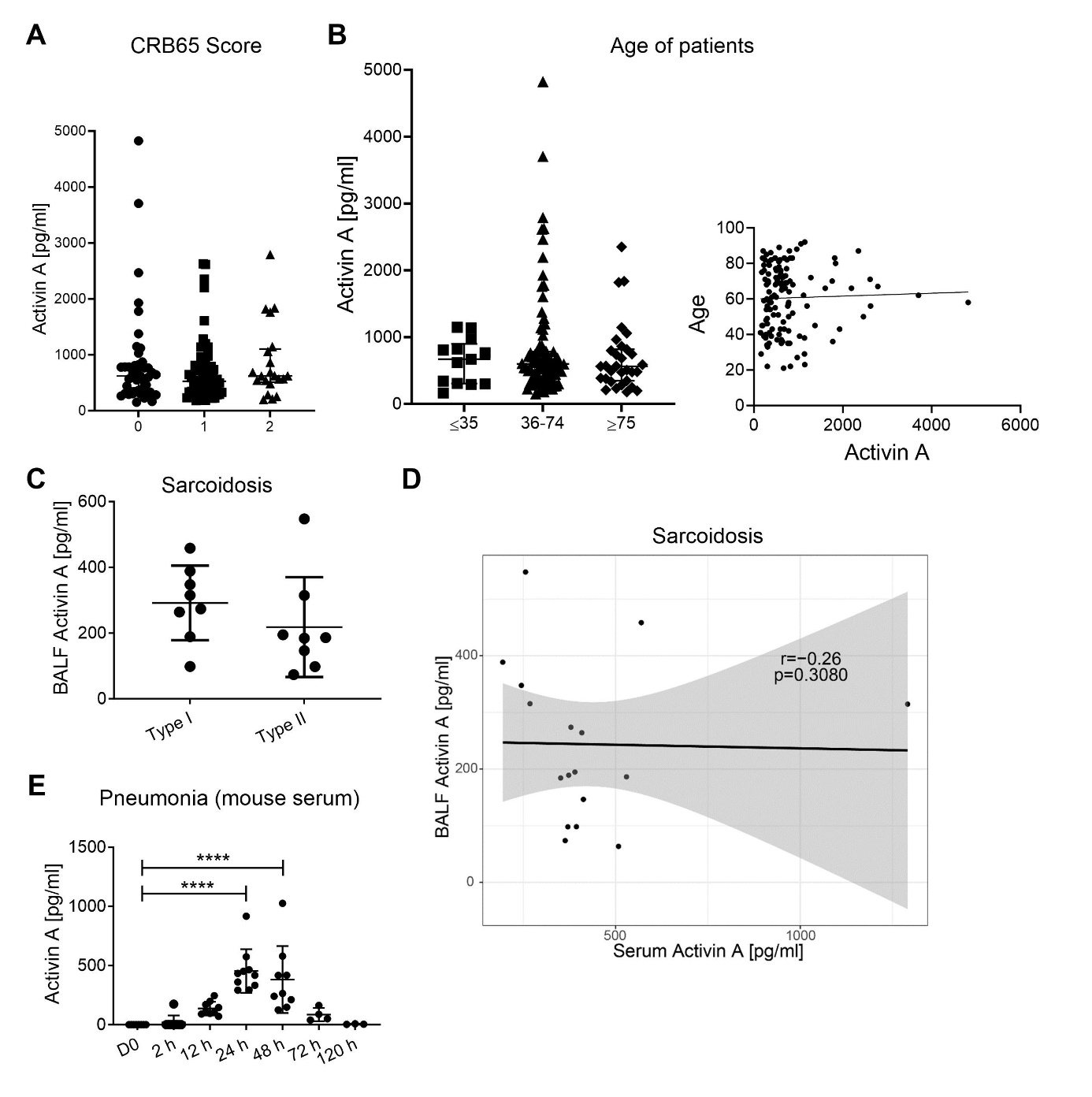


**Fig. S3.** Supplementary data on activin A in pneumonia and sarcoidosis. A) Serum activin A levels in relation to CRB65 score. The CRB65 score is a clinical score used to estimate the severity of community-acquired pneumonias that gives on point each to: Confusion, Rate of respiration ≥30, Blood pressure: systolic BP <90 mmHg or diastolic BP ≤60 mmHg, and age: 65 and over. B) Serum activin A in pneumonia patients stratified into age groups, and lack of association of age and activin A in pneumonia patients. C) BALF activin A levels in sarcoidosis patients stratified according to X ray type. Type I: bilateral hilar adenopathy; Type 2: additionally, involvement of pulmonary parenchyma. D) Association of serum and BALF activin A in sarcoidosis patients. Pearson’s correlation with t-test was calculated and results are accompanied by linear regression. E) Activin A levels in serum from mice infected with *Streptococcus pneumoniae*. ****, *P*<0.0001. Mice were infected with 5 × 10^6^ CFU of *Spn* serotype 2 and BALF was collected at indicated timepoints after infection. Control mice were sham-infected with PBS. Timepoints post 24 hours show only survivors of infection. Statistical significance was calculated using the Mann-Whitney test in the case of two groups and the Kruskal-Wallis test with Dunn’s multiple comparison test in the case of multiple groups.

**
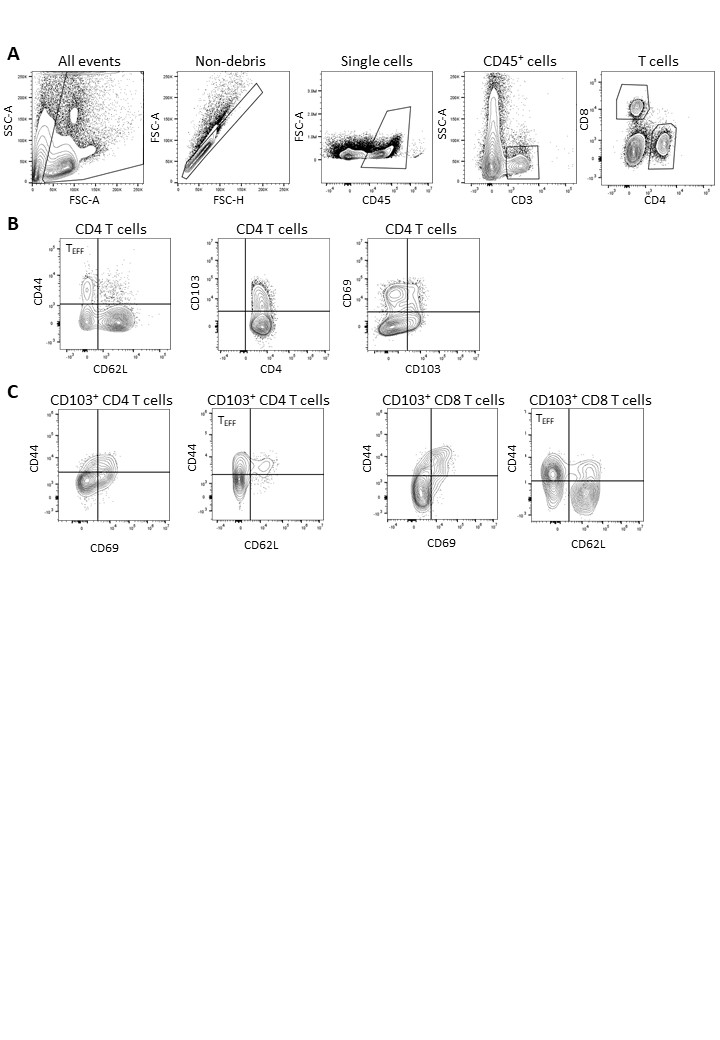
**

**Fig. S4.** Gating of CD103^+^ T cell populations in the lungs. Data were analyzed in FlowJo. A) Gating of CD4 and CD8 T cell populations in lungs at day 14 post infection. B) Gating of T_EFF_ (CD44^+^CD62L^-^), CD103^+^ and CD103^+^CD69^+^ CD4 T cell populations in the lungs. The same gating strategy was used for CD8 T cells. C) Phenotype of CD103^+^ CD4 and CD8 T cells showing CD44, CD69, CD44 and CD62L expression.


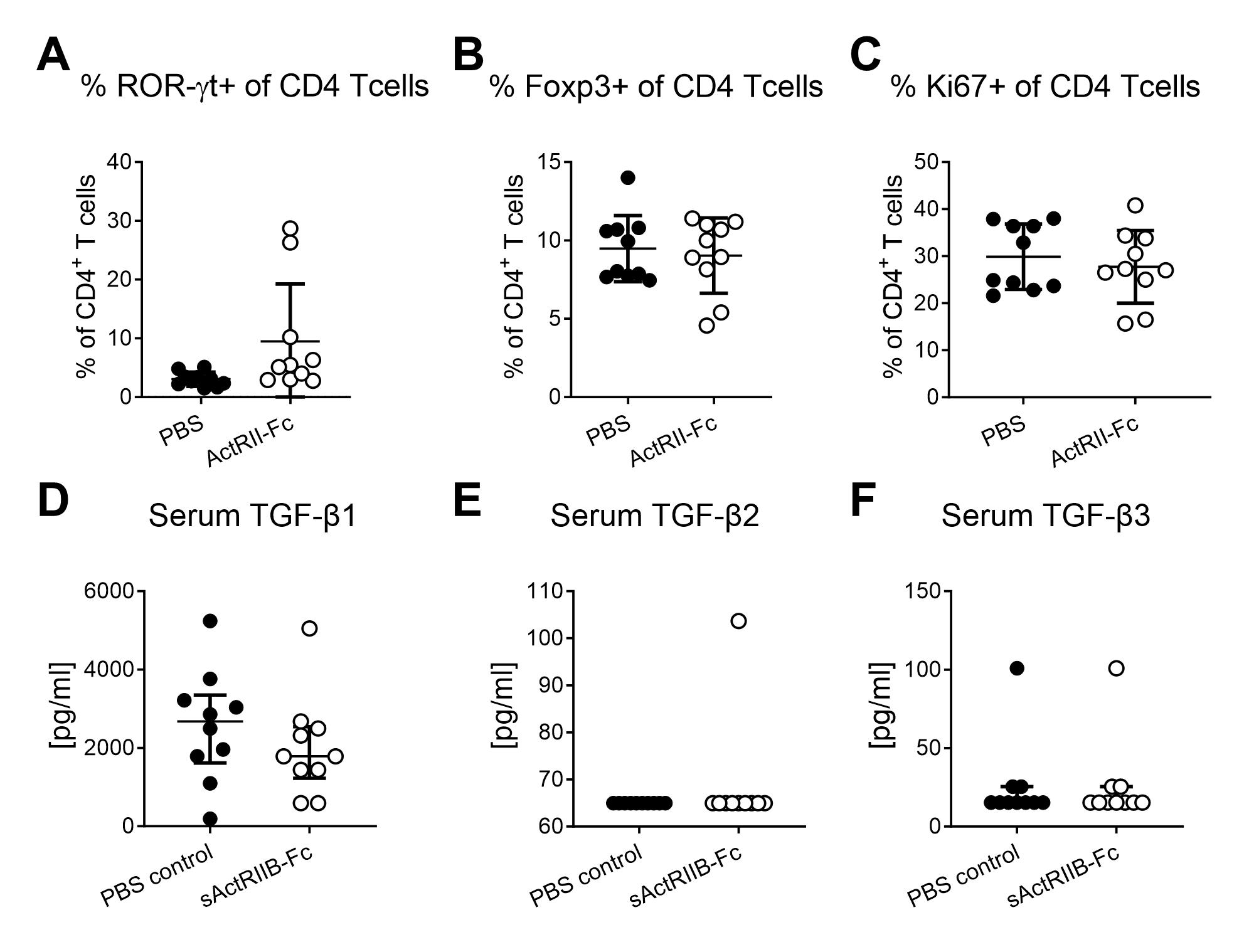


**Fig. S5.** Effect of ActRIIB-Fc treatment on transcription factor expression and TGF-beta levels during *Mtb* infection. A) Percentages of ROR-γt^+^ CD4 T cells. B) Percentage of Foxp3^+^ CD4 T cells. C) Percentages of Ki67^+^ CD4 T cells. D) Serum levels of TGF-β 1. E) Serum levels of TGF-β 2. F) Serum levels of TGF-β3. Graphs show mean with standard deviation, n = 10, pooled from two experiments with n = 5 per group. Statistical significance was calculated using the unpaired t test. *, *P*<0.05; **, *P*<0.005.

**
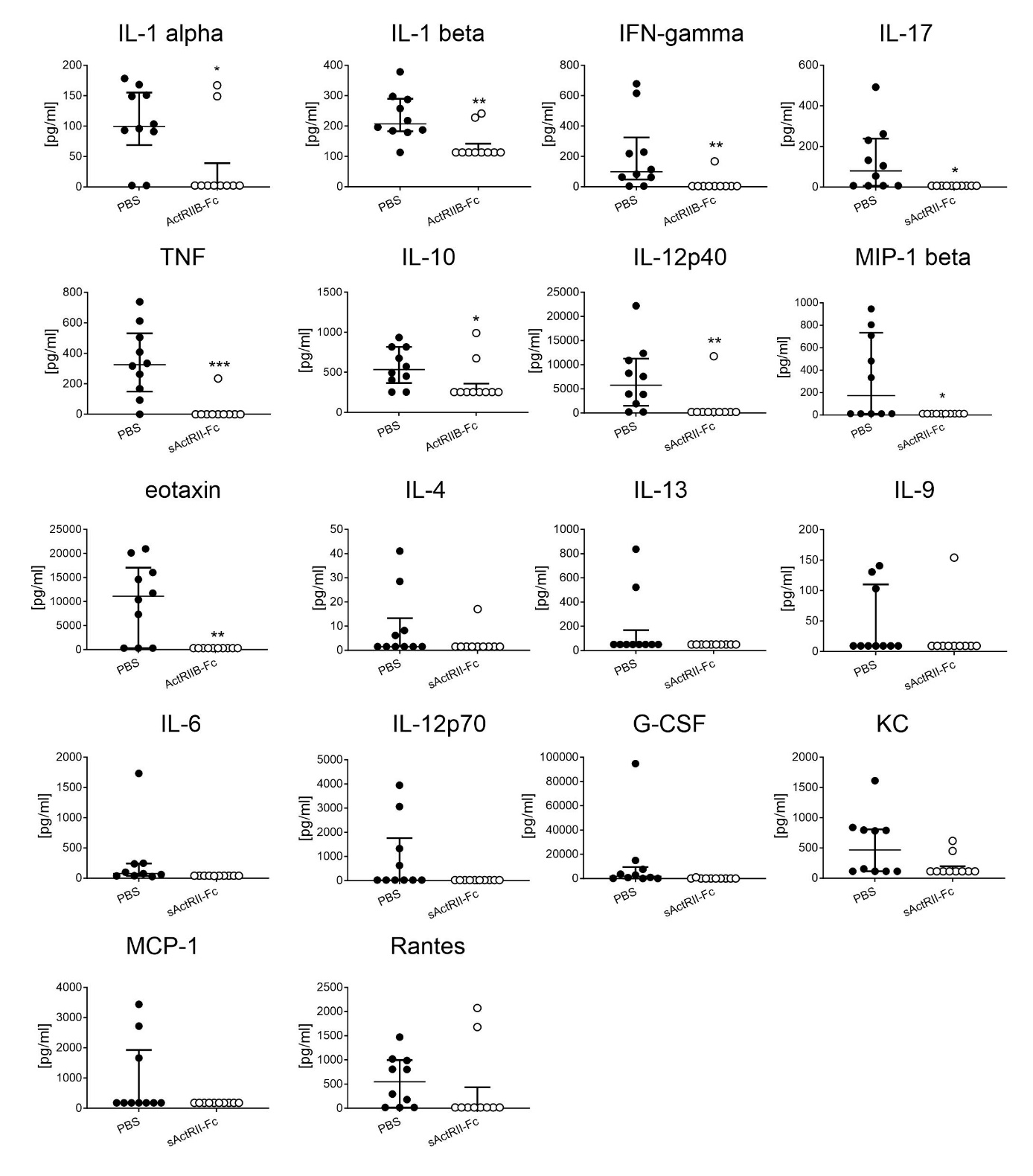
**

**Fig. S6.** Effect of administration of ActRIIB-Fc on the serum cytokine profile during *Mtb* H37Rv infection in mice. Responses were measured at D14 post infection (n = 10 per group). Cytokines and chemokines were measured in serum using the Bio-Plex Mouse Cytokine 23-plex Assay. IL-2, IL-3, IL-5, GM-CSF and MIP-1α were not detected in any samples. Statistical significance was calculated using the Mann-Whitney test. *, P<0.05; **, P<0.01; ***; P<0.001. IL-1 alpha: *P* = 0.025; IL-1 beta: *P* = 0.006; IFN-gamma: *P* = 0.003; IL-17: *P* = 0.011; TNF: *P* = 0.0003; IL-10: *P* = 0.036; IL-12p40: *P* = 0.004; MIP-1 beta: *P* = 0.033; eotaxin: *P* = 0.003.


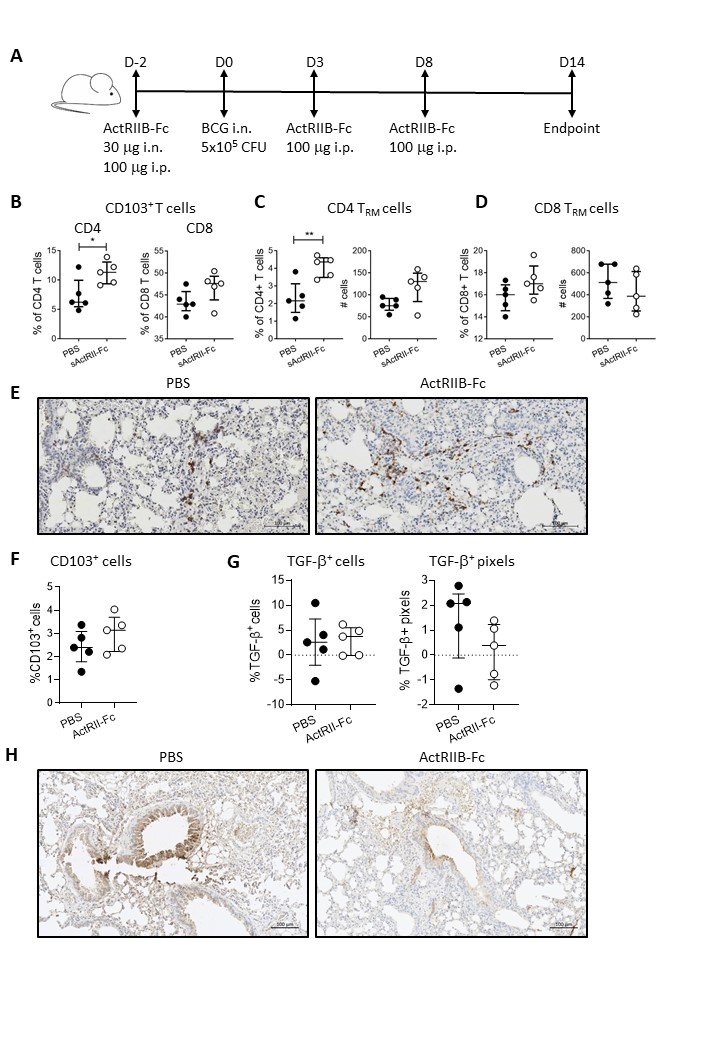


**Fig. S7.** The effect of ActRIIB-Fc treatment on T resident memory cell populations in BCG-vaccinated mice. A) Experimental schedule. ActRIIB-Fc was administered i.n. + i.p. two days prior to i.n. BCG vaccination and i.p. once a week thereafter. Cell populations in the lungs were analyzed by flow cytometry at day 14 p.i.. B) Percentages of CD103^+^ cells among CD4 and CD8 T cells. *, P = 0.039. C) Percentages and numbers of CD103^+^CD69^+^ T_RM_ cells. **, P = 0.007. D) Percentages and numbers of CD103^+^CD69^+^ T_RM_ cells E) Staining of CD103^+^ cells in lung tissue sections. F) Quantification of CD103^+^ cells in lung tissue sections. G) Quantification of TGF positive cells and pixels in lung tissue sections. Data were normalized to negative control sections. H) TGF-β staining of lung tissue sections and negative control sections. Statistical significance was calculated using the Mann-Whitney test.

**REFERENCES**

1. Falk A, O'Connor J, Pratt P, Webb W, Wier J, Wolinsky E. 1969. Diagnostic standards and classification of tuberculosis. Bull Natl Tuberc Respir Dis Assoc:68-76.

2. Ralph AP, Ardian M, Wiguna A, Maguire GP, Becker NG, Drogumuller G, Wilks MJ, Waramori G, Tjitra E, Sandjaja, Kenagalem E, Pontororing GJ, Anstey NM, Kelly PM. 2010. A simple, valid, numerical score for grading chest x-ray severity in adult smear-positive pulmonary tuberculosis. Thorax 65:863-9.

3. Welte T, Suttorp N, Marre R. 2004. CAPNETZ-community-acquired pneumonia competence network. Infection 32:234-8.

4. Bauer TT, Ewig S, Marre R, Suttorp N, Welte T, Group CS. 2006. CRB-65 predicts death from community-acquired pneumonia. J Intern Med 260:93-101.

5. Kruger S, Ewig S, Giersdorf S, Hartmann O, Suttorp N, Welte T, German Competence Network for the Study of Community Acquired Pneumonia Study G. 2010. Cardiovascular and inflammatory biomarkers to predict short- and long-term survival in community-acquired pneumonia: Results from the German Competence Network, CAPNETZ. Am J Respir Crit Care Med 182:1426-34.

6. Schnerch J, Prasse A, Vlachakis D, Schuchardt KL, Pechkovsky DV, Goldmann T, Gaede KI, Muller-Quernheim J, Zissel G. 2016. Functional Toll-Like Receptor 9 Expression and CXCR3 Ligand Release in Pulmonary Sarcoidosis. Am J Respir Cell Mol Biol 55:749-757.

7. Anonymous. 1999. Statement on sarcoidosis. Joint Statement of the American Thoracic Society (ATS), the European Respiratory Society (ERS) and the World Association of Sarcoidosis and Other Granulomatous Disorders (WASOG) adopted by the ATS Board of Directors and by the ERS Executive Committee, February 1999. Am J Respir Crit Care Med 160:736-55.

8. Scadding JG. 1961. Prognosis of intrathoracic sarcoidosis in England. A review of 136 cases after five years' observation. Br Med J 2:1165-72.

9. Myllarniemi M, Tikkanen J, Hulmi JJ, Pasternack A, Sutinen E, Ronty M, Lepparanta O, Ma H, Ritvos O, Koli K. 2014. Upregulation of activin-B and follistatin in pulmonary fibrosis - a translational study using human biopsies and a specific inhibitor in mouse fibrosis models. BMC Pulm Med 14:170.

10. Apostolou E, Stavropoulos A, Sountoulidis A, Xirakia C, Giaglis S, Protopapadakis E, Ritis K, Mentzelopoulos S, Pasternack A, Foster M, Ritvos O, Tzelepis GE, Andreakos E, Sideras P. 2012. Activin-A overexpression in the murine lung causes pathology that simulates acute respiratory distress syndrome. Am J Respir Crit Care Med 185:382-91.

11. Hulmi JJ, Oliveira BM, Silvennoinen M, Hoogaars WM, Ma H, Pierre P, Pasternack A, Kainulainen H, Ritvos O. 2013. Muscle protein synthesis, mTORC1/MAPK/Hippo signaling, and capillary density are altered by blocking of myostatin and activins. Am J Physiol Endocrinol Metab 304:E41-50.

12. Chiu CS, Peekhaus N, Weber H, Adamski S, Murray EM, Zhang HZ, Zhao JZ, Ernst R, Lineberger J, Huang L, Hampton R, Arnold BA, Vitelli S, Hamuro L, Wang WR, Wei N, Dillon GM, Miao J, Alves SE, Glantschnig H, Wang F, Wilkinson HA. 2013. Increased muscle force production and bone mineral density in ActRIIB-Fc-treated mature rodents. J Gerontol A Biol Sci Med Sci 68:1181-92.
